# EzySCR: A free and easy tool for scoring event‐related skin conductance responses in the first, second, and third interval latency windows

**DOI:** 10.1111/psyp.14686

**Published:** 2024-10-16

**Authors:** Luke J. Ney, Jorge L. Pardo, Ottmar V. Lipp

**Affiliations:** ^1^ School of Psychology and Counselling Queensland University of Technology Brisbane Queensland Australia; ^2^ Centre for Accident Research & Road Safety Queensland University of Technology Brisbane Queensland Australia

**Keywords:** electrodermal activity, fear conditioning, skin conductance responding

## Abstract

Skin conductance is a commonly used physiological measure during psychology experiments, such as during fear conditioning. Methods for scoring skin conductance responses (SCRs) are highly heterogeneous, though most researchers agree that manually inspected scores provide the highest quality data when compared to most available fully automated scoring methods. However, manual scoring is extremely time‐consuming. We developed a semi‐automated scoring program that reduces the time required to process SCR data at a level of quality akin to manual scoring. In contrast to all previous scoring programs, our program enables scoring of first interval response (FIR), second interval response (SIR), and third interval response (TIR) SCRs. Using interclass correlation coefficients (ICCs), Bland–Altman plots and Pareto analysis, we show here that our method is highly reliable and produces data that are almost identical to data that are manually scored and scored using LEDALAB. This software is very easy to use and is freely available to download and modify. We expect that this software will be helpful in reducing the time required to produce high quality FIR, SIR, and TIR skin conductance data for psychology researchers around the world.

## Abstract 2

Existing automated methods for scoring skin conductance responses (SCRs) without manual inspection leave ambiguity whether these methods are scoring SCRs accurately. Our program automatically scores SCRs and allows rapid manual inspection of multiple response intervals for each event. This program provides an option for researchers to produce high quality SCR data in a time‐efficient manner. The program is free, easy to use, and is opensource. The program is fully validated against manually scored data and between independent raters.

## 1 INTRODUCTION

Skin conductance is a physiological measure of autonomic activity that is often used to make inferences about psychological states and processes. Event‐related skin conductance responses (SCRs) reflect sudomotor nerve‐induced changes in the electrical conductivity of the skin, which is mediated by increased sudomotor‐controlled sweat secretion from endocrine sweat glands (Boucsein, 2012; Wallin, 1981). Sudomotor nerves are part of the sympathetic nervous system and are under the control of brain regions such as the hypothalamus, hippocampus, and amygdala, meaning that some indication of neural activity during behavioral tests can be ascertained from peripheral SCRs (Ojala & Bach, 2020; Tranel & Damasio, 1994), though it is not currently clear what type of neural activity SCRs reflect. SCRs have a distinct response function that shows a gradual increase to a peak, followed by a long tail while the skin conductance level (SCL) returns to baseline (Bach et al., 2010). Therefore, experiments that use skin conductance but have short inter‐trial intervals will incur overlapping SCRs, which may make differentiating responses difficult during scoring and may underestimate the size of a response to a later stimulus due to incomplete response recovery (Alexander et al., 2005).

Fear conditioning paradigms frequently use SCR as a primary outcome measure to infer learning and memory processes occurring in anticipation of threatening stimuli. Fear conditioning experiments are used both to understand how a fear association is acquired as well as how safety memories are formed (called extinction). Fear acquisition and extinction are key concepts underlying the gold‐standard treatments for anxiety and trauma‐related disorders, where patients are repeatedly exposed to a feared stimulus or situation so that an extinction memory can be developed (Craske et al., 2014; Zuj & Norrholm, 2019). A typical fear conditioning experiment consists of multiple phases, habituation, acquisition, and extinction. During habituation, the neutral, to‐be‐conditioned stimuli (CSs) are presented alone to reduce orienting that any novel stimulus will elicit. During acquisition, one neutral stimulus is repeatedly paired with an unconditioned stimulus (US; e.g., an electric shock). The formerly neutral stimulus (now a conditioned stimulus or CS+) will come to invoke a threat response by itself, whereas CS that are not paired with the US during acquisition will not (called a CS−). SCRs are used in fear conditioning experiments to index physiological arousal following the CS+ and SCRs during an extinction phase—where the US never follows the CS+—indicate whether a participant has successfully learned that the CS+ is no longer a threatening cue (Lipp, 2006). In this way, extinction is said to model exposure therapy and the fear conditioning paradigm can thus be used to determine what individual and experimental factors might improve or impair extinction (Craske et al., 2014).

Multiple methods exist for the scoring of SCRs in fear conditioning paradigms. Traditionally, SCRs were scored manually without computer assistance, though it is unlikely that this is still performed in any modern laboratory internationally. Computer‐assisted manual scoring methods are still frequently used and involve scorers checking each event (i.e., CS onset) during an experiment on a computer and selecting the minimum (trough) and maximum (peak) values of each SCR (Boucsein, 2012; Kuhn et al., 2022; Levinson & Edelberg, 1985). Notably, this method involves visual confirmation of an SCR following an event, which contrasts with automated methods of SCR scoring that usually do not require that a scored SCR is a true SCR response. In particular, baseline‐corrected SCR scoring methods—which are to our knowledge always automated—score responses following events by subtracting the average SCL prior to the event onset from the maximum value during the event (Orr et al., 2000; Pineles et al., 2009). A significant limitation of this method of SCR scoring is that it cannot accurately determine whether the maximum value during an event reflects an actual SCR or just a tonic increase (or decrease) in SCL (Sjouwerman et al., 2022). In cases where the baseline SCL is higher than the maximum value during the event, a negative value is scored, which is by definition not an SCR given the SCR's distinct response function. Moreover, scoring SCRs based on tonic changes in SCL does not reflect sudomotor nerve bursts but rather slow changes in arousal originating from the reticular formation of the brainstem (Boucsein, 2012), which is unlikely to be involved in higher‐level psychological processes. Despite these issues, baseline correction remains one of the most popular methods for scoring SCRs in fear conditioning (Kuhn et al., 2022).

Computer‐automated scoring methods that use the trough‐to‐peak scoring method have also been developed (Green et al., 2014; Storm et al., 2000). “Autonomate” uses the MATLAB interface to import files representing skin conductance and will automatically score SCRs following event markers using parameters such as detection slope (i.e., the minimum SCL gradient that can be considered to be an SCR), detection length (i.e., the minimum duration of the SCL increase that can be considered to be an SCR), and so on, which define the required response function for the program to identify an SCR following an event (Green et al., 2014). The program then provides trough‐to‐peak amplitude values that can be manually checked and adjusted by a scorer. Further, the SCR identification parameters are user‐adjustable, meaning that if a user notices that SCRs are not being identified they may optimize the peak detection of the program. However, the program requires the event markers to be of a certain format and only accepts files that are saved through the BIOPAC Acknowledge software, which is not universally used. Autonomate is occasionally used in fear conditioning research, though based on available evidence baseline‐correction methods and computer‐assisted manual scoring are more frequently used (Kuhn et al., 2022). Scoring methods that attempt to mathematically model the neural responses underlying skin conductance data are available, such as the PsPM and deconvolution methods such as LEDALAB (Bach et al., 2010, 2011; Benedek & Kaernbach, 2010b), and these are occasionally used for fear conditioning research (Kuhn et al., 2022). These approaches estimate scores using a peripheral model that infers sudomotor nerve activity as a proxy of sympathetic activity. The PsPM and LEDALAB approaches therefore do not produce SCRs as output but rather inferred (i.e., modeled) estimates of the time series of sudomotor nerve activity that SCRs should reflect. However, these programs require some mathematical and computer expertise, which may be a reason that they are not frequently adopted despite being reported to result in better discrimination of CS+ from CS− responses compared to trough‐to‐peak scoring (Staib et al., 2015), as well as being fully automated.

A common limitation of all of the above automated approaches is that they do not offer the ability to score SCRs in multiple latency windows following an event onset. Beginning in the 1970s, it has been argued that SCRs following a CS should be scored into first interval (FIR), second interval (SIR), and third interval (TIR) responses (Prokasy et al., 1973; Prokasy & Ebel, 1967). The FIR commences between 1 and 4 s following CS onset (Sjouwerman & Lonsdorf, 2019) and is believed to reflect the orienting response towards the presented stimulus (Öhman, 1983). The SIR occurs from 4 s until 1 s post‐CS, the timing of which will vary depending on the duration of the CS (Prokasy et al., 1973). The SIR is believed to reflect an anticipatory response as potential reinforcement of the US draws nearer, assuming that the US has been presented immediately following CS offset in previous trials (Öhman, 1983). The TIR reflects a response to the presence or absence of the US and is measured 1–4 s following the US presentation. Both the FIR and SIR showed conditionability, and it is debated whether they show enough concordance to warrant analysis using entire interval (EIR) scoring techniques, where the largest response throughout both the FIR and SIR latency windows is scored as the SCR (Luck & Lipp, 2016; Pineles et al., 2009; Prokasy et al., 1973). On one hand, it is argued that FIR and SIR are statistically independent, reflect different processes, and should not be scored together (Prokasy et al., 1973). On the other hand, it has been argued more recently that scoring an EIR shows a close relationship with responses from both the FIR and SIR (Pineles et al., 2009), though in this case the EIR were scored using a baseline correction. More recently it was demonstrated that analysis of the FIR and SIR separately may uncover effects not covered by the EIR alone, particularly using conditioning paradigms involving manipulations that will result in enhanced orienting to both CS+ and CS− (Luck & Lipp, 2016).

Given all of the reasons outlined above, many fear conditioning laboratories still prefer to use computer‐assisted manual SCR scoring over fully automated methods, despite the significantly higher time and resources required (Kuhn et al., 2022). In the current manuscript, we describe a program that we developed that automatically scores trough‐to‐peak SCRs into FIR, SIR, and TIR intervals following an event marker. Our program allows manual inspection and adjustment of the scores provided by the program. The program is open source, has optimizable parameters, and accepts .mat files rather than only acknowledge files. We tested the reliability of the scores against manually scored data as well as peak‐to‐trough scores provided by LEDALAB. Finally, we identified sources of discrepancies as well as directions for future improvements and areas of focus in the program.

## 2 METHODS

### 2.1 Program design

The EzySCR interface is a stand‐alone web application that requires local installation using Anaconda Distribution and Git. It was developed using Python (v3.8), Jupyter notebooks (Kluyver et al., 2016), IpyWidgets (IpyWidgets Dev Team, 2015), and Voilà (QuantStack, 2019). The EzySCR Jupyter notebook is publicly available on GitHub (https://github.com/jorgpg5/EzySCR). A manual for how to install and use the program is included in the Supplementary Material to this article.

Prior to analysis, the data recorded at 1000 Hz were pre‐processed. A high‐pass filter with a Hamming window and a frequency cut‐off of 0.5 Hz was applied to the data. SCRs were segmented into windows of L secs following each stimulus. The data were then down‐sampled to 50 Hz using a convenience method for frequency conversion and resampling of time series provided by the Pandas library (The Pandas Dev Team, 2020). The algorithm identified the rises of candidate SCRs by searching for the highest point, surrounded by points lower by a threshold “X” on both sides. The default threshold value “X” is defined as 0.02 μS (Green et al., 2014). The algorithm uses a difference of 0.02 μS between a peak and its surroundings to declare it as a peak. The trough is calculated with the same logic. The algorithm returns the “index” that contains the peak and trough values. Some laboratories will wish to use different peak detection thresholds. This parameter can be changed on the loading screen by changing the default value in the “Detection Threshold” box from 0.02 to whatever value a user wishes to use.

**FIGURE 1:**
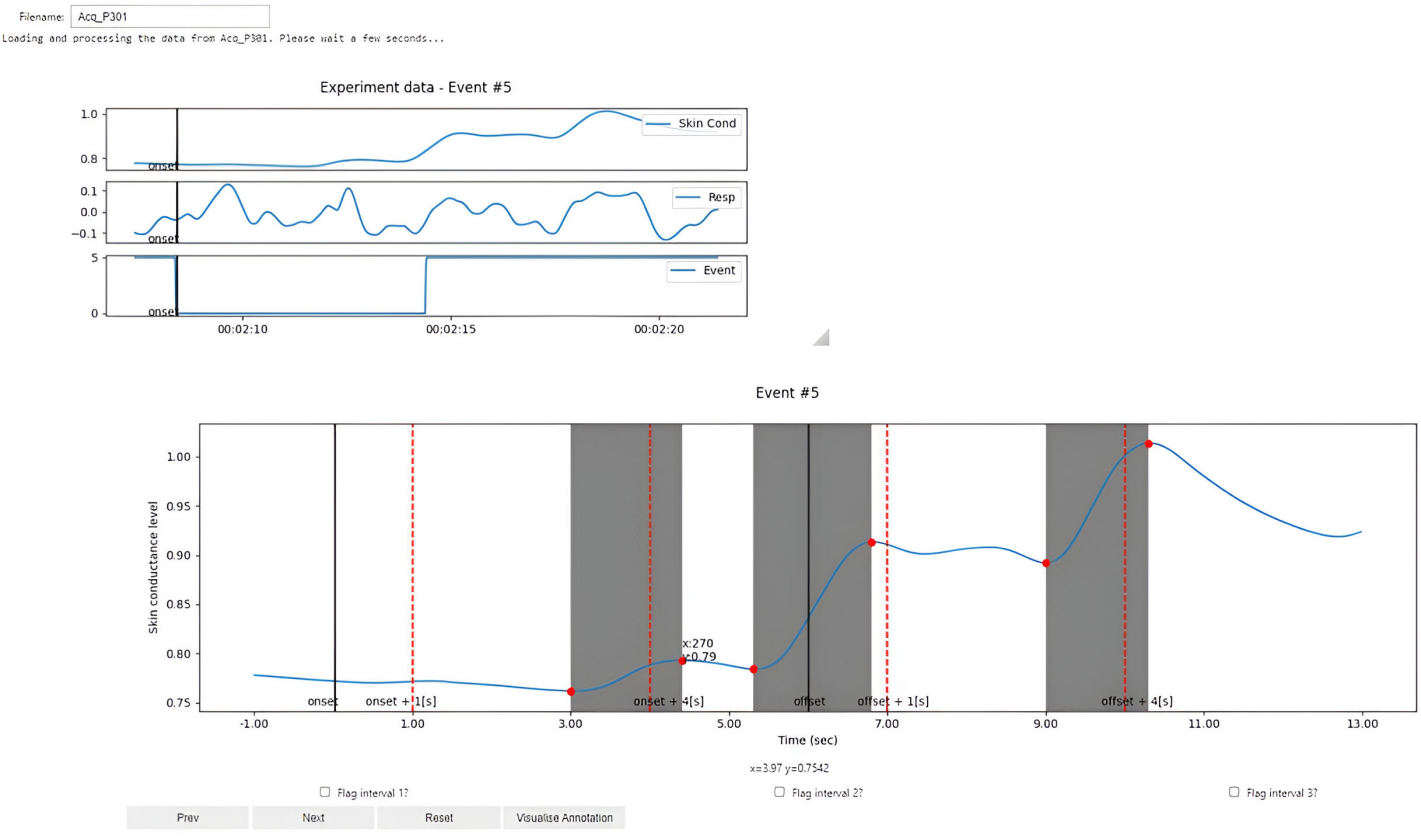
The EzySCR interface. Skin Cond = skin conductance channel, Resp = respiration channel, Event = Event Marker Channel (i.e., conditioned stimulus presentations). Users enter their datafile name in the “Filename” box and the program will automatically score the SCRs for each event (otherwise known as each trial). Skin conductance, respiration, and event markers are visualized in smaller figures at the top, and the skin conductance from the three intervals is presented in the larger figure. Users can adjust the suggested scores (numerically stated at the bottom of the page) by dragging the red dots to highlight the correct skin conductance peaks, which are indicated by the shaded sections of the figure. The red dotted vertical lines indicate the first, second, and third interval boundaries. The solid black vertical lines indicate the start and end of the CS onset. They can then go to the next or previous trials by clicking “Next” or “Prev”, or optionally reset the trial if they have made a mistake or encountered an error. Event #5 in this figure refers to the CS trial being the 5th measured event (in this case the 5th trial).

The EzySCR interface shows the candidate SCRs (Figure 1). A candidate is provided for each interval of the SCRs, including the FIR, SIR, and TIRs. The interface shows and annotates the candidates for FIR (between 1 and 4 s following CS onset), SIR (from 4 s until 7 s post‐CS onset), and TIR (1–4 s following CS offset). Each event from the Event channel of a user's data is scored. In our data, the Event channel only includes CS trials, though in another laboratory's Event channels other events during the experiment may be recorded. Users must specify their trial order for events in their experiment (e.g., CS+ trials, CS− trials) post‐data scoring.

The peak detection algorithm revolves around the idea of a sliding window, scanning the data efficiently in chunks to identify local maxima (peaks) and minima (troughs) within a specified interval. This method inherently filters out minor fluctuations, ensuring that only significant variations are considered. Once detected, each peak and trough first undergoes a threshold‐based validation process: a peak is confirmed only if it is at least “delta” units higher than a subsequent trough, and vice versa for troughs. From the validated set, the algorithm retains only the most prominent peak and the deepest trough, discarding other candidates. If the algorithm does not identify any valid values, it provides “default” values, hinting at the need for potential manual annotation. Users can fine‐tune the algorithm's results to enhance adaptability by dragging markers to the correct positions. Additionally, a user interface option allows marking specific intervals as “missing/artifact” when necessary. The interface automatically saves the annotations in a CSV file containing all the annotated data.

Since the program is semi‐automated and the peak scores are visualized, no pre‐screening of data is necessary (e.g., to filter bad responses). Secondly, the interface already has the option to down‐sample uploaded data. This means that data does not need to be pre‐processed prior to analysis using the program.

### 2.2 Software validation

To validate that our scoring program shows concordance to manual SCR scoring, we re‐analyzed subsets of data from two recently completed studies in our laboratory (*n* = 20 each was randomly selected). These data had previously been scored manually by a trained research assistant (see details below). We trained a second research assistant to manually score these data a second time to test for inter‐scorer reliability. Next, L.J.N. and a trained research assistant each independently scored the data using EzySCR.

The studies were two fear conditioning experiments conducted in our laboratory at the Queensland University of Technology between 2021 and 2022. Both experiments consisted of a simple differential paradigm involving a single CS+ and a CS− presented during habituation, acquisition, and extinction phases. The habituation, acquisition, and extinction phases consisted of 4, 8, and 24 trials of each CS, respectively. In both experiments, the CSs were either a picture of a bird, a frog, or a fish. CS duration was 6 s and intertrial intervals (CS offset to CS onset) jittered randomly between 10 and 14 s. During acquisition, the CS+ was followed by US 100% of the time. In Study 1, the US was always a series of three 2 ms “unpleasant, but not painful” electric shocks (spaced 16 ms apart and perceived as one discrete stimulus), the intensity of which was calibrated prior to the experiment to each participants' perception of this criterion and was delivered via a concentric electrode to the dominant forearm. In Study 2, half of participants received the shock, and the other half instead heard a loud, female scream (IADS sound number 277; Bradley & Lang, 2007), which lasted for 6 s at a maximum amplitude of 90 dBA (Ney et al., 2023).

Note that the habituation and acquisition phases were recorded in the same data file and were consequently scored together. There was a short break between acquisition and extinction where participants rated CS pleasantness in both experiments (resulting in separate data recordings), but there was no such break between habituation and acquisition.

### 2.3 Manual scoring

Two research assistants were trained by L.J.N. and O.V.L. to score FIR, SIR, and TIRs manually using Acknowledge v3.9.1 (BIOPAC). Acknowledge visualizes the skin conductance trace and time‐locked event markers and has functionality that allows a scorer to tabulate SCRs relative to the event markers on a trial‐by‐trial basis. Research assistants were trained to identify SCRs and differentiate them from artifacts in the skin conductance trace. SCR identification was based on the principles and parameters outlined in the Methods Program Design section, and skin conductance artifacts were defined as fluctuations that did not meet these criteria or were immediately preceded by unusual respiration activity, which constituted probably bodily movement of the participant. Non‐responses during a latency interval (same definition as above) were scored as 0. The research assistants had their first five data files cross‐checked against previously scored data to ensure consistency of scoring against more experienced scorers.

### 2.4 LEDALAB scoring

LEDALAB v3.4.9 was downloaded and used to score our SCR data as a comparison to EzySCR (Benedek & Kaernbach, 2010a, 2010b). Data was down sampled to 50 Hz with the down sample type set at “Factor Mean”. Continuous Decomposition Analysis was selected to analyze the data, with Analyze and Optimize functions run before executing the analysis. The Export Event‐Related Action function was used to obtain SCR peaks within specified timing brackets. As the imported event markers represented visual stimulus offset, the SCR intervals relative to event (start—end) were as follows: −7 to −4 (for the 1–4 SIR), −4 to −1 (for the 4–7 SIR), and −1 to −2 (for the 7–10 SIR). SCR amplitude minimum was set at 0.02 and the *z*‐score values box was checked.

Due to the files always importing an event marker at 0.01 s of the file, the sample had to be cut to exclude this first marker. Otherwise, the negative time window parameter, i.e., −7 to −4 would not run in Ledalab. Therefore, the Cut Data function was used to encapsulate all data except for the first event marker, excluding it from analysis.

### 2.5 Statistical analyses

Agreement interclass correlation coefficients (ICCs, McGraw & Wong, 1996) were used to assess the relationship between independent scorers using the EzySCR program. Similarly, agreement ICCs were calculated to test the concordance of EzySCR with manual scoring as well as with LEDALAB scoring. All of these analyses were conducted using one‐way random effects models and were used to test the average SCR of each participant as well as trial‐by‐trial concordance across participants. Bland–Altman plots (Bland & Altman, 1986) were used to visualize the agreement between raters using EzySCR as well as between manual and EzySCR methods. Bland Altman plots allow evaluation of agreement between individual raters by plotting the differences between two scoring methods and the confidence interval of these differences. Notably, this method of analysis does not provide interpretation of whether the observed limits are acceptable. The mean difference between raters or methods and its 95% Confidence Interval is plotted in this manuscript, which allows easy visualization of the number of scores that are substantially different between raters or between methods. Scores that were significantly different between raters or methods were compiled and qualitatively investigated using the Pareto method (Gougeon, 2008). This involved summation and qualitative assessment of the reasons for discrepancy between raters or between methods.

## 3 RESULTS

**FIGURE 2:**
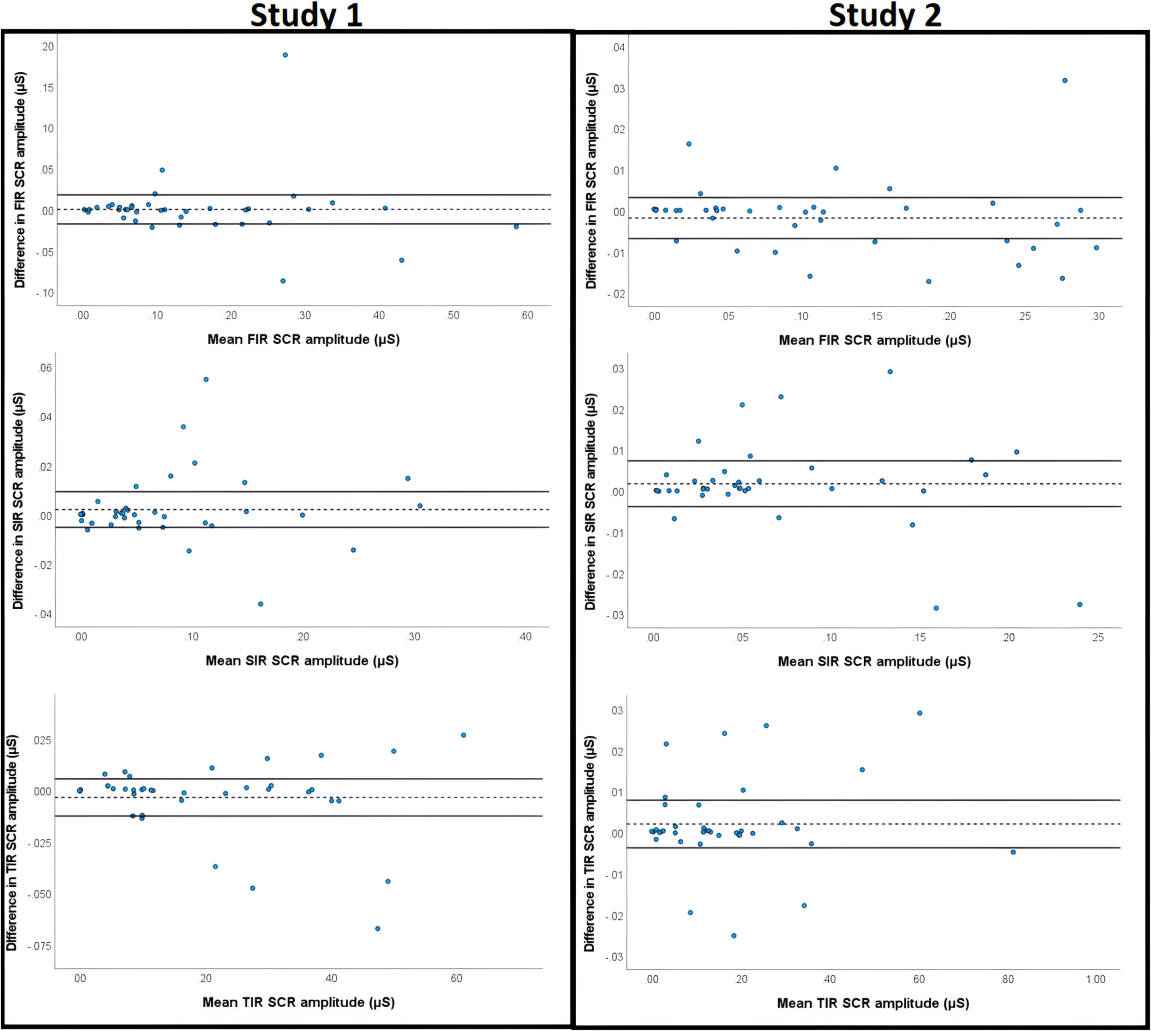
Bland–Altman plots for inter‐rater reliability using EzySCR. Left column is Study 1 and the right column is Study 2. Row 1 is FIR, row 2 is SIR and row 3 is TIR. The mean (dotted line) with 95% confidence intervals (thick line) are depicted. Scores outside these lines are outside the expected discrepancy between raters at a 95% confidence interval level. These discrepancies are also plotted on the *x*‐axis against the mean SCR amplitude scored by the two raters, which did not reveal any systematic bias of higher discrepancies with higher or lower SCR amplitudes. These discrepant scores were later subjected to Pareto analysis to determine the causes of discrepant scores. FIR, first interval response; SCR, skin conductance response; SIR, second interval response; TIR, third interval response.

**FIGURE 3:**
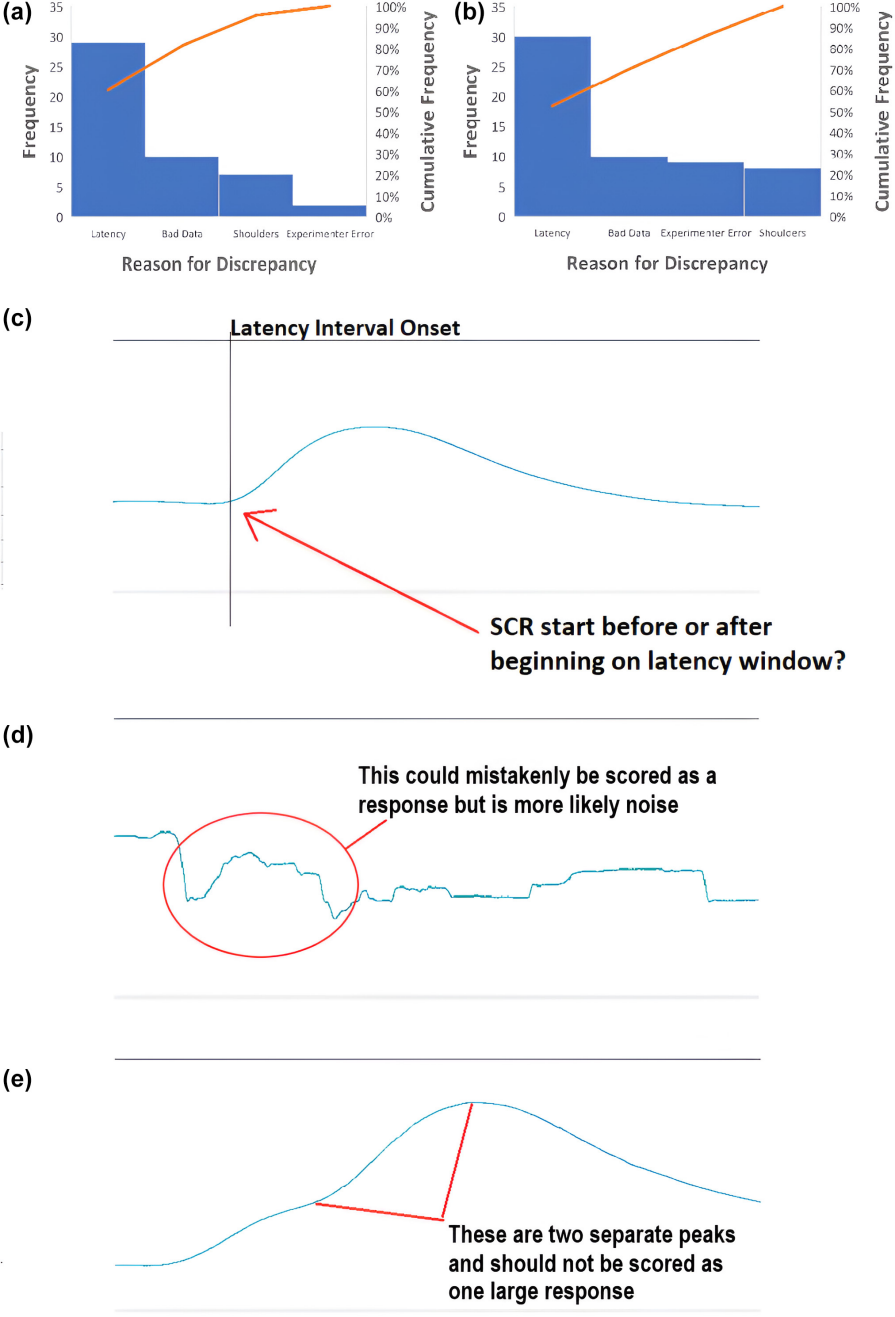
Pareto analysis and corresponding examples of reasons for discrepancies between scores. (a) These reasons between raters who only used EzySCR. (b) The reasons for discrepancies of scores identified when EzySCR was compared to manual scoring. (c) An example of a latency interval discrepancy. Panel D shows an example of poor‐quality skin conductance data. (E) An example of a skin conductance peak shoulder that resulted in a discrepancy between scores.

Analyses using ICC showed that inter‐rater reliability using EzySCR was exemplary for both studies, both experimental phases, and all SCR latency intervals (Table 1). This was true for both averaged SCRs as well as trial‐by‐trial SCRs when tested between independent raters. In addition to Table 1, we also found that ICC agreement between CS+ and CS− trials was almost equivalent, with CS− being slightly, but not significantly, higher, ΔICC = 0.01. Bland–Altman plots (Figure 2) illustrate this high level of agreement between independent raters using EzySCR. Relative to the number of agreeing scores, outliers were uncommon (10%–20% of data points—consisting of averaged trials for each participant—contained on average 1.2 discrepant values across all trials). Outlying values were summed and analyzed using Pareto analyses (Figure 3a). These analyses revealed that the source of discrepancies between raters was overwhelmingly disagreement, in which latency interval SCRs belonged, followed by poor quality SCR data, peak shoulders, and experimenter error. Examples of latency interval issues, bad SCR data, and peak shoulders are visualized in Figure 3. Experimenter error consisted of two cases where scorers failed to record a peak where there was a clear SCR.

**TABLE 1:** Interclass correlation coefficients show high agreement between raters who scored skin conductance response data across multiple phases and latency intervals using EzySCR.

	SCR latency interval	Phase	ICC	*F* statistic
Average scores				
Study 1	FIR	Hab/Acq	0.993	142.46***
		Extinction	0.998	567.18***
	SIR	Hab/Acq	0.979	47.43***
		Extinction	0.995	209.90***
	TIR	Hab/Acq	0.996	267.49***
		Extinction	0.995	185.51***
Study 2	FIR	Hab/Acq	0.997	398.01***
		Extinction	0.935	15.33***
	SIR	Hab/Acq	0.991	116.30***
		Extinction	0.994	177.07***
	TIR	Hab/Acq	0.999	1287.74***
		Extinction	0.996	265.14***
Trial‐by‐trial				
Study 1	FIR	Hab/Acq	0.961	25.88***
		Extinction	0.964	27.88***
	SIR	Hab/Acq	0.946	18.65***
		Extinction	0.970	33.24***
	TIR	Hab/Acq	0.990	105.07***
		Extinction	0.987	75.30***
Study 2	FIR	Hab/Acq	0.986	69.19***
		Extinction	0.978	45.20***
	SIR	Hab/Acq	0.944	17.77***
		Extinction	0.973	37.30***
	TIR	Hab/Acq	0.998	425.63***
		Extinction	0.960	24.77***

Abbreviations: FIR, first interval response; ICC, interclass correlation coefficient; SCR, skin conductance response; SIR, second interval response; TIR, third interval response.

*** *p* < .001. Hab/Acq refers to data scored from the combined Habituation and Acquisition phases of the experiment.

Inter‐rater reliability of manual scoring was also high, though not as high as inter‐rater reliability using EzySCR (Table 2), despite an equivalent amount of time training assistants on how to use the scoring techniques. The manual scoring also required a significantly longer time to compile and clean compared to compilation of EzySCR data, due to the higher propensity for mistakes made by scorers.

**TABLE 2:** Interclass correlation coefficients show adequate agreement between raters who scored skin conductance response data across multiple phases and latency intervals manually.

	SCR latency interval	Phase	ICC	*F* statistic
Average scores				
Study 1	FIR	Hab/Acq	0.996	252.81***
		Extinction	0.968	31.32***
	SIR	Hab/Acq	0.992	119.59***
		Extinction	0.918	12.23***
	TIR	Hab/Acq	0.994	162.25***
		Extinction	0.976	41.07***
Study 2	FIR	Hab/Acq	0.988	85.41***
		Extinction	0.993	137.48***
	SIR	Hab/Acq	0.970	33.81***
		Extinction	0.967	30.30***
	TIR	Hab/Acq	0.993	134.22***
		Extinction	0.933	15.01***
Trial‐by‐trial				
Study 1	FIR	Hab/Acq	0.804	5.10***
		Extinction	0.834	6.02***
	SIR	Hab/Acq	0.617	2.61***
		Extinction	0.845	6.45***
	TIR	Hab/Acq	0.801	5.03***
		Extinction	0.906	10.66***
Study 2	FIR	Hab/Acq	0.986	69.19***
		Extinction	0.915	11.74***
	SIR	Hab/Acq	0.944	17.77***
		Extinction	0.908	10.91***
	TIR	Hab/Acq	0.998	425.63***
		Extinction	0.972	36.14***

*Note*: Hab/Acq refers to data scored from the combined Habituation and Acquisition phases of the experiment.

Abbreviations: FIR, first interval response; ICC, interclass correlation coefficient; SCR, skin conductance response; SIR, second interval response; TIR, third interval response.

*** *p* < .001.

**FIGURE 4:**
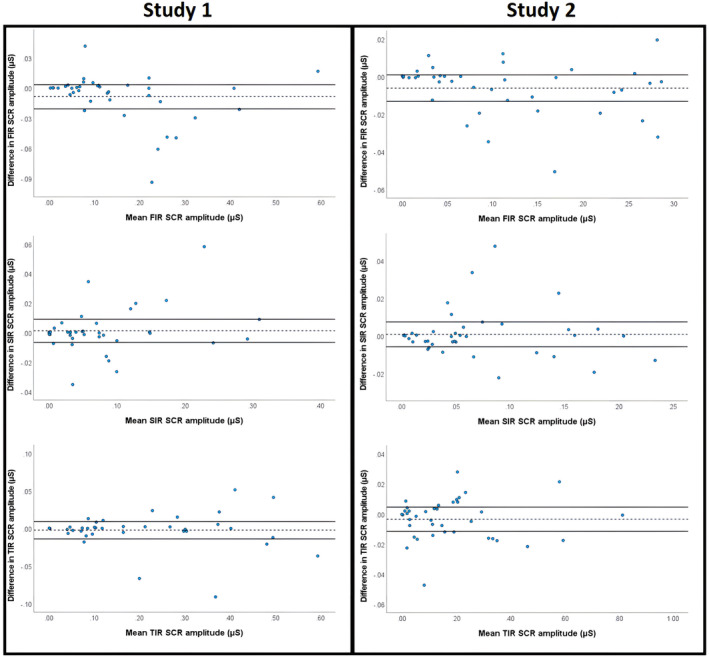
Bland–Altman plots for agreement between EzySCR and manual scoring. Left column is Study 1 and the right column is Study 2. Row 1 is FIR, row 2 is SIR and row 3 is TIR. The mean (dotted line) with 95% confidence intervals (thick line) are depicted. Scores outside these lines are outside the expected discrepancy between rates at a 95% confidence interval level. These discrepancies are also plotted on the *x*‐axis against the mean SCR amplitude scored by the two raters, which did not reveal any systematic bias of higher discrepancies with higher or lower SCR amplitudes. These discrepant scores were later subjected to Pareto analysis to determine the causes of discrepant scores. FIR, first interval response; SCR, skin conductance response; SIR, second interval response; TIR, third interval response.

Agreement between SCRs scored manually and using EzySCR was established using ICCs (Table 3). We found excellent concordance between the scoring methods, both overall and across individual trials. Bland–Altman plots (Figure 4) illustrate the distribution of the average scores between scoring methods, with the average scores between raters used for each method. Outlying values were again subjected to Pareto analysis, which revealed similar patterns to those revealed between raters using EzySCR. Primarily, discrepancies were driven by latency interval disagreement, followed by bad SCR data quality, experimenter error, and SCR peak shoulders.

**TABLE 3:** Interclass correlation coefficients show high agreement between raters who scored skin conductance response data across multiple phases and latency intervals manually when compared with scoring using EzySCR.

	SCR latency interval	Phase	ICC	*F* statistic
Average scores				
Study 1	FIR	Hab/Acq	0.995	204.81***
		Extinction	0.972	35.76***
	SIR	Hab/Acq	0.992	119.59***
		Extinction	0.993	147.62***
	TIR	Hab/Acq	0.993	134.31***
		Extinction	0.994	154.07***
Study 2	FIR	Hab/Acq	0.992	118.06***
		Extinction	0.996	267.98***
	SIR	Hab/Acq	0.979	47.43***
		Extinction	0.998	658.52***
	TIR	Hab/Acq	0.998	536.36***
		Extinction	0.996	267.87***
Trial‐by‐trial				
Study 1	FIR	Hab/Acq	0.980	51.19***
		Extinction	0.958	23.78***
	SIR	Hab/Acq	0.953	21.22***
		Extinction	0.967	29.89***
	TIR	Hab/Acq	0.995	216.05***
		Extinction	0.975	40.75***
Study 2	FIR	Hab/Acq	0.842	6.33***
		Extinction	0.978	45.64***
	SIR	Hab/Acq	0.977	44.17***
		Extinction	0.908	10.91***
	TIR	Hab/Acq	0.998	425.63***
		Extinction	0.993	141.62***

*Note*: Hab/Acq refers to data scored from the combined Habituation and Acquisition phases of the experiment.

Abbreviations: FIR, first interval response; ICC, interclass correlation coefficient; SCR, skin conductance response; SIR, second interval response; TIR, third interval response.

*** *p* < .001.

**FIGURE 5:**
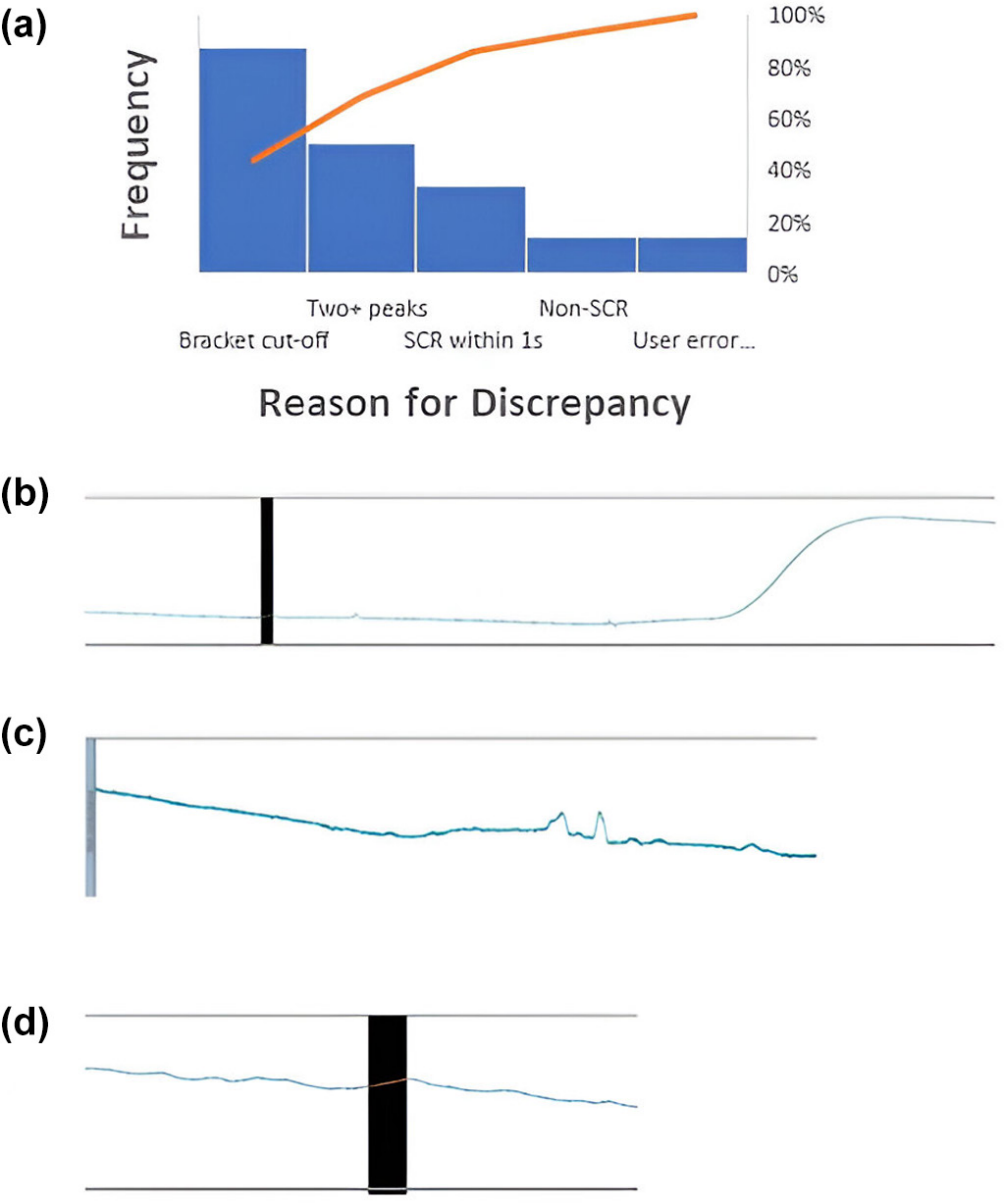
Reasons for discrepancies between LEDALAB and EzySCR. (a) The Pareto analysis of the discrepancies, where Bracket cut‐off means SCRs in LEDALAB were underestimated because LEDALAB did not measure the SCR after the latency window finished. Two+ peaks means that LEDALAB summed two or more SCRs as one SCR. SCR within 1 s means that SCRs were counted by LEDALAB despite beginning within 1 s of stimulus onset. Non‐SCR means that LEDALAB scored a SCR despite it being due to noise. (b, c) Non‐SCR fluctuations that were scored as SCRs by both LEDALAB and EzySCR but were removed by research assistants during manual inspection using EzySCR. (d) An example of a discrepancy where both programs scored a SCR, but it may, or may not have, been removed during manual inspection due to being an edge case of what a SCR should look like.

Finally, the agreement between SCRs scored using LEDALAB and using EzySCR was established using ICCs (Table 4). We found excellent concordance between the scoring methods, both overall and across individual trials. An exception to this was the TIRs for both the trial‐by‐trial and averaged scores during acquisition. Outlying values were again assessed. Discrepancies of the TIRs during acquisition were driven by the fact that LEDALAB did not score SCRs that peaked after the prescribed interval, suggesting that this program should not be used for TIRs. Discrepancies in other data were driven mostly by LEDALAB erroneously scoring noise artifacts as SCRs, summing multiple SCRs together, or scoring SCRs that began within 1 s of event onset (Figure 5). Some discrepancies were also due to user error when using EzySCR. It should be noted that there were some edge cases where it was debatable whether a true SCR occurred. These cases were often scored by the EzySCR algorithm but removed after manual inspection by the trained scorers.

**TABLE 4:** Interclass correlation coefficients show high agreement between skin conductance response data across multiple phases and latency intervals scored using LEDALAB when compared with scoring using EzySCR.

	SCR latency interval	Phase	ICC	*F* statistic
Average scores				
Study 1	FIR	Hab/Acq	0.976	42.28***
		Extinction	0.995	208.41***
	SIR	Hab/Acq	0.989	90.87***
		Extinction	0.905	10.51***
	TIR	Hab/Acq	−.071	0.93
		Extinction	0.939	16.40***
Trial‐by‐trial				
Study 1	FIR	Hab/Acq	0.920	12.55***
		Extinction	0.982	55.79***
	SIR	Hab/Acq	0.925	13.39***
		Extinction	0.827	5.77***
	TIR	Hab/Acq	0.333	1.50***
		Extinction	0.901	10.14***

*Note*: Hab/Acq refers to data scored from the combined Habituation and Acquisition phases of the experiment.

Abbreviations: FIR, first interval response; ICC, interclass correlation coefficient; SCR, skin conductance response; SIR, second interval response; TIR, third interval response.

*** *p* < .001.

## 4 DISCUSSION

Here we describe the development and validation of a semi‐automated SCR scoring method. In contrast to previous programs, our method permits scoring of FIR, SIR, and TIR electrodermal event‐related responses. Our program—EzySCR—shows high inter‐rater reliability and strong agreement with manually‐scored responses. Discrepancies in scores between EzySCR and manual scoring were infrequent and predictable and were largely associated with disagreement between raters as to whether peaks belonged to different latency intervals, but only in cases where it was unclear when an SCR peak began. Discrepancies in scores between EzySCR and LEDALAB were also infrequent, except for during TIR responses where LEDALAB did not score values that peaked after the TIR window. Our program is easy to use and will provide researchers with the ability to decrease the amount of time needed to manually score event‐related SCR data without sacrificing data quality.

Manual scoring of SCR data has long been the gold‐standard for quantification of electrodermal data. However, manual scoring of SCR data is time‐consuming and prone to error. Previous research groups have developed programs that enable automated scoring of both event‐related and spontaneous SCRs (Bach et al., 2009; Benedek & Kaernbach, 2010a; Green et al., 2014; Pineles et al., 2009). However, in some cases (Bach et al., 2009; Benedek & Kaernbach, 2010a; Pineles et al., 2009) these scoring methods do not permit manual adjustment of SCRs within the scoring program. This means that SCRs do not undergo manual inspection and are not subject to researcher scrutiny when decided which electrodermal activity is event‐related. In particular, baseline correction methods are highly likely to produce scores that do not reflect event‐related SCRs, due to the absence of a peak detection algorithm. Despite this limitation, many fear conditioning laboratories continue to use baseline correction (Kuhn et al., 2022), which is likely due to the ease of implementation. In contrast, deconvolution methods (Bach et al., 2009; Benedek & Kaernbach, 2010a) are complicated theoretically and can be difficult to implement, which has resulted in slow uptake in the fear conditioning community. Autonomate, developed by the LaBar laboratory (Green et al., 2014) provides a validated alternative, with automated trough‐to‐peak SCR scoring that can be later manually adjusted by the researcher through visual inspection. While this method is easy to use, it does not allow scoring of FIR, SIR, and TIRand does not currently allow for unconventional event marker types.

In the current study, we developed a program that can score SCRs automatically within three latency intervals following an event. The program then allows users to manually adjust the suggested scores, greatly reducing the amount of time required to completely score SCRs at a level comparable to manual scoring. Trained research assistants scored habituation, acquisition, and extinction SCR data from two studies using both manual scoring (with Acknowledge v3.9.1) and EzySCR. Agreement between raters within and between these scoring methods was tested using ICCs. We found that EzySCR ICCs were exemplary, which suggests that raters independently recorded almost identical scores. The inter‐rater agreement of manual scoring was slightly lower, but still high. This suggests that EzySCR produces slightly higher inter‐rater agreement. Agreement between EzySCR and manual scoring was very high, suggesting that results produced by EzySCR are similar to those produced manually. This finding is important because EzySCR cuts down the time required to score SCRs by at least half, requires less training, and does not require as many data collating steps when compared to SCRs produced by manual scoring.

Our analysis was able to detect some causes of discrepancies between raters when using EzySCR. Discrepancies between raters could be resolved in future by including interaction functionality that shows when a slope is increasing so that peak onset can be more easily observed by research staff. Alternatively, additional training could be provided to staff to ensure that decision‐making is uniform. Further, improvement of inter‐rater reliability could be achieved by better pre‐processing of poor‐quality SCR data (e.g., data affected by hardware issues during recording) prior to scoring.

When compared to LEDALAB, EzySCR shows comparable accuracy, though we found that LEDALAB had a tendency to retain non‐SCR fluctuations and was not able to accurately score TIR responses. To score all three intervals, LEDALAB also needed to be run three times per participant, in contrast to EzySCR's one time per participant. So, in this case, if researchers are looking to only score FIR responses, LEDALAB—while potentially slightly less accurate due to no manual screening stage—may save researchers time, so long as the skin conductance data does not have frequent and large artifacts. If researchers are looking to score multiple intervals, EzySCR may be a time efficient method, and if scoring the TIR, will be more accurate.

EzySCR has been demonstrated here to be an efficient and time‐effective method of maintaining a high SCR data quality. Future improvements to the program include improving the peak detection algorithm and customizable options that suit different datasets and researchers' requirements. On Windows laptops, the computer must be plugged into its charger for the program to function at the highest speed. This issue persists even if the Windows laptop power management settings are changed, so Windows laptops need to be charged for the highest efficiency. This issue does not exist on Mac computers. However, the program is already fully operational and is available on GitHub. It does not require any expert knowledge to use and accepts .mat files, which can be saved from multiple common physiology software (e.g., LabChart, Acknowledge). A related general limitation of the software is that it currently only accepts .mat files and it is not possible to immediately import all files from all software programs. If a user finds that their software format is not compatible with EzySCR then the users can contact the authors so that a specific import pathway can be developed for their use. Data on SCR software recording programs that we extracted from a recent systematic review (Wang et al. (2024), data on software recording programs not published) indicates that over 50% of the fear conditioning field likely uses BIOPAC with Acknowledge software, with many other programs making up smaller shares of the field, including LabChart. In this case, the majority of recorded SCR data will be compatible with the current options available with EzySCR. EzySCR has successfully been tested in two laboratories in North America, one in Europe, and three in Australia. Most issues are due to conflicting formatting of data files and are easily fixable by our team. Clearly, the method is still more time‐consuming than automated scoring, but manual inspection of the SCR data gives the assurance that the data processing is of the highest possible quality. Finally, future directions for EzySCR also include greater customizability of parameters upon program start‐up, such as marker size.

In conclusion, we developed an efficient and easy‐to‐use scoring program permitting manual‐quality scoring of SCRs in FIR, SIR, and TIR. This method is free to use and modify and can be downloaded from GitHub. The method is highly reliable and produces between inter‐rater agreement compared to manual scoring, while maintaining a high level of data quality. This program should make SCR scoring easier for fear conditioning laboratories around the world.

## AUTHOR CONTRIBUTIONS

**Luke J. Ney:** Conceptualization; data curation; formal analysis; investigation; methodology; project administration; supervision; validation; visualization; writing – original draft; writing – review and editing. **Jorge L. Pardo:** Investigation; methodology; project administration; software; writing – original draft; writing – review and editing. **Ottmar V. Lipp:** Conceptualization; funding acquisition; investigation; resources; supervision; writing – review and editing.

## FUNDING INFORMATION

This work was supported by the Australian Research Council (DP180100869) and the National Health and Medical Research Council (GNT1156490).

## Supporting information


Data S1.
