# Supplementary material for: EzySCR: A free and easy tool for scoring event‐related skin conductance responses in the first, second, and third interval latency windows
Source: Psychophysiology. 2024 Oct 16;61(12):e14686. doi: 10.1111/psyp.14686 (PMC11579223; doi:10.1111/psyp.14686)
Supplement: Supplementary file 1 — Data S1. [file PSYP-61-e14686-s001.docx]

**EzySCR Manual**

v1.1

Dr Luke J Ney^1^, Mr Jorge Pardo Gaytan^1,2^, and Professor Ottmar V Lipp^1^

^1^School of Psychology and Counselling, Queensland University of Technology, Australia

^2^Centre for Accident Research & Road Safety, Queensland University of Technology, Australia

**Purpose**

Skin conductance responding is a frequently used outcome measure in human Pavlovian fear conditioning, though the scoring of this data using appropriate methods can be time-consuming. EzySCR allows semi-automated processing of event-related skin conductance response (SCR) data across multiple latency interval windows. By default, and assuming a 6-second conditional stimulus (CS) duration, these latency windows include the First Interval (1-4 seconds following CS onset), Second Interval (4-7 seconds following CS onset) and Third Interval (7-10 seconds following CS onset, a.k.a. 1-4 seconds following CS offset) Responses. These windows, as well as the CS duration, are user-adjustable, as described below. This manual is intended to provide a comprehensive explanation of how to install and use the EzySCR program. Any additional information concerning its use can be attained by contacting the authors (contact details provided below).

Previous work has developed automated methods for scoring single SCR intervals, though in practice it is always unknown whether these methods are scoring SCRs accurately without manual inspection. Our program automatically scores SCRs and allows rapid manual inspection of multiple response intervals for each event. In our experience, EzySCR reduced scoring time by up to 90% while maintaining the same high data-quality produced by fully manual scoring. This is a substantial improvement upon previous methods and will help researchers produce high quality SCR data in a time-efficient manner. The program is free and easy to use. It is available for download from Github (https://github.com/jorgpg5/EzySCR) and is also opensource, which permits users to make adjustments for the needs of their own laboratory. It was developed using Python (v3.8), Jupyter notebooks (Kluyver et al., 2016), ipyWidgets (IpyWidgets Dev Team, 2015), the Pandas library (The Pandas Dev Team, 2020), and Voilà (QuantStack, 2019). The program is also regularly updated, so if you have issues with any aspects of functionality or can suggest improvements, please get in touch with the research team. We have fully validated this method against manually scored data and between independent raters. This validation data is reported in the manuscript (under review at Psychophysiology).

This work was funded by the Australian Research Council (DP180100869) and the National Health and Medical Research Council (GNT1156490). We would like to thank Matthew O’Donohue, Tynneille Mulder, Elisabeth Muller, Yi Wang, Kobi Eales, and Sarah Olsson for their research assistance during the project.


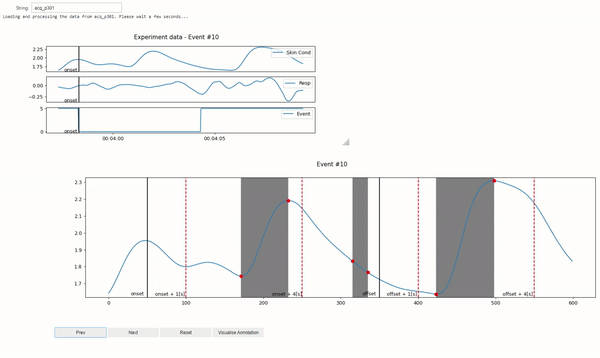


***Figure S1.*** *The EzySCR interface.*

**Algorithm**

The algorithm identified the rises of candidate skin conductance responses by searching for the highest point, surrounded by points lower by a threshold ”X” on both sides. The default threshold value “X” is defined as 0.02 µS (Green et al., 2014). The algorithm requires a difference of 0.02 µS between a peak and its surroundings to declare it as a peak. As well the trough is calculated with the same logic. The algorithm returns the “index” that contains the peak and trough values. Once detected, each peak and trough first undergoes a threshold-based validation process: a peak is confirmed only if it’s at least “delta” units higher than a subsequent trough, and vice versa for troughs. From the validated set, the algorithm retains only the most prominent peak and the deepest trough, discarding other candidates. If the algorithm does not identify any valid values, it provides “default” values, hinting at the need for potential manual annotation. Users can fine-tune the algorithm’s results to enhance adaptability by dragging markers to the correct positions. Additionally, a user interface option allows marking specific intervals as “missing/artifact” when necessary. The interface automatically saves the annotations in a CSV file containing all the annotated data.

Some laboratories will wish to use different peak detection thresholds. This parameter can be changed on the loading screen by changing the default value in the “Detection Threshold” box from 0.02 to whatever value a user wishes to use.

**Installation and Setup**

Currently, the only way to install EzySCR is by cloning the program from the Github repository and executing the software as a notebook in a local browser through an Anaconda prompt. This is slightly more complicated than installing a program as a professional package, though allows advanced users to modify the software code to allow full customisability of the package. As many users will not be familiar with Github or Anaconda, we have created a video demonstration of the software installation to compliment the instructions in this manual. The video can be accessed here: <https://youtu.be/n47qZ0FdE6g>

**NOTE:** The video version of this manual uses an older version of the program, “semiautomated_annotation”. This archived title for the program uses a different Github pathway with the name <https://github.com/jorgpg5/semiautomated_annotation.git> rather than <https://github.com/jorgpg5/EzySCR.git>. Except for this detail, the video can be followed in exact detail.

To install this software, we'll need Anaconda and Git. If you already have them, you can skip to the **User Guide** section. Keep in mind that although installation is not as easy as it could be, you only need to install once.

We recommend users to have admin rights for installing the software. Although we have not encountered any issues arising from file permission issues during testing at two universities in Australia and one in Germany, there is a possibility that your institution may block installation of the program. If this is the case, then you will need to either request admin rights or rights to install Python, Git, and/or Anaconda from your IT administrator to complete installation.

**1.** Please download and install [Anaconda](https://www.anaconda.com/products/distribution) (click link and follow). Please do not check or uncheck any installation options.

[
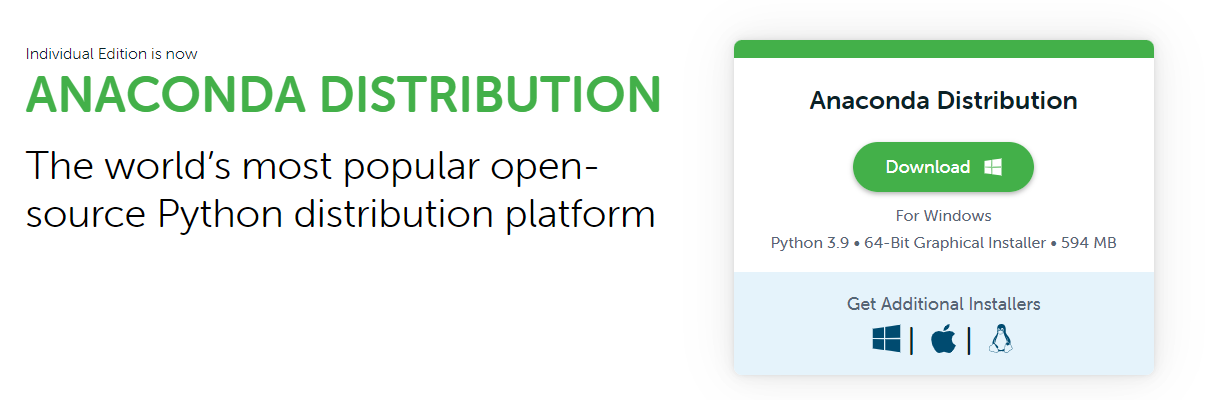
](https://user-images.githubusercontent.com/70129680/182262876-d91d2d50-b4dc-44db-a0aa-1f91cf8c1146.png)

***Figure S2.*** *The Anaconda download portal.*

**2.** Next, please download and install [Git](https://git-scm.com/downloads)  (click link and follow):

[
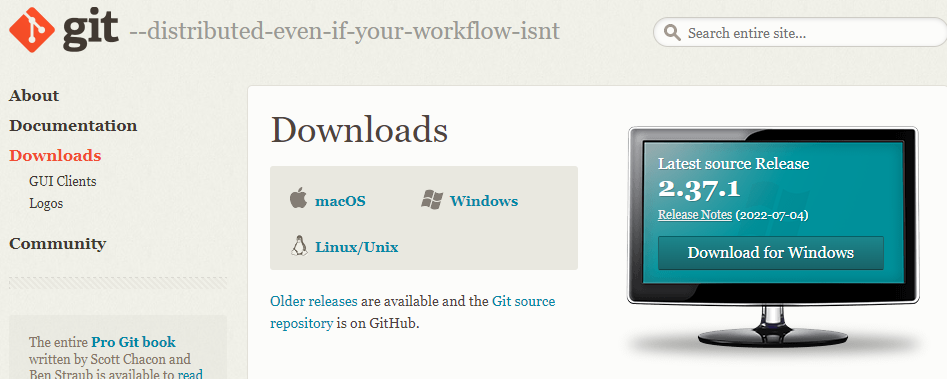
](https://user-images.githubusercontent.com/70129680/182263939-001858b8-8782-42c3-9122-fb90833f80f2.png)

***Figure S3.*** *The git download portal.*

**3.** Go to the folder where you want save the interface software. Open a git bash (right click on the folder and select Git Bash here). NOTE: this should be saved in a file location that has no spaces in it’s name, e.g., “C:/Users/ney_luke/” is OK, but “C:/Users/ney luke/” will not work correctly.

[
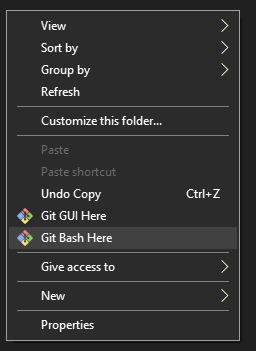
](https://user-images.githubusercontent.com/70129680/182267804-5e0ee31f-08a1-4f18-a833-ebb2b297d5fe.png)

***Figure S4.*** *Right-click in your destination folder to create a Git Bash.*

**4.** We will clone this repository. Copy the location of this repository (i.e., https://github.com/jorgpg5/EzySCR.git):


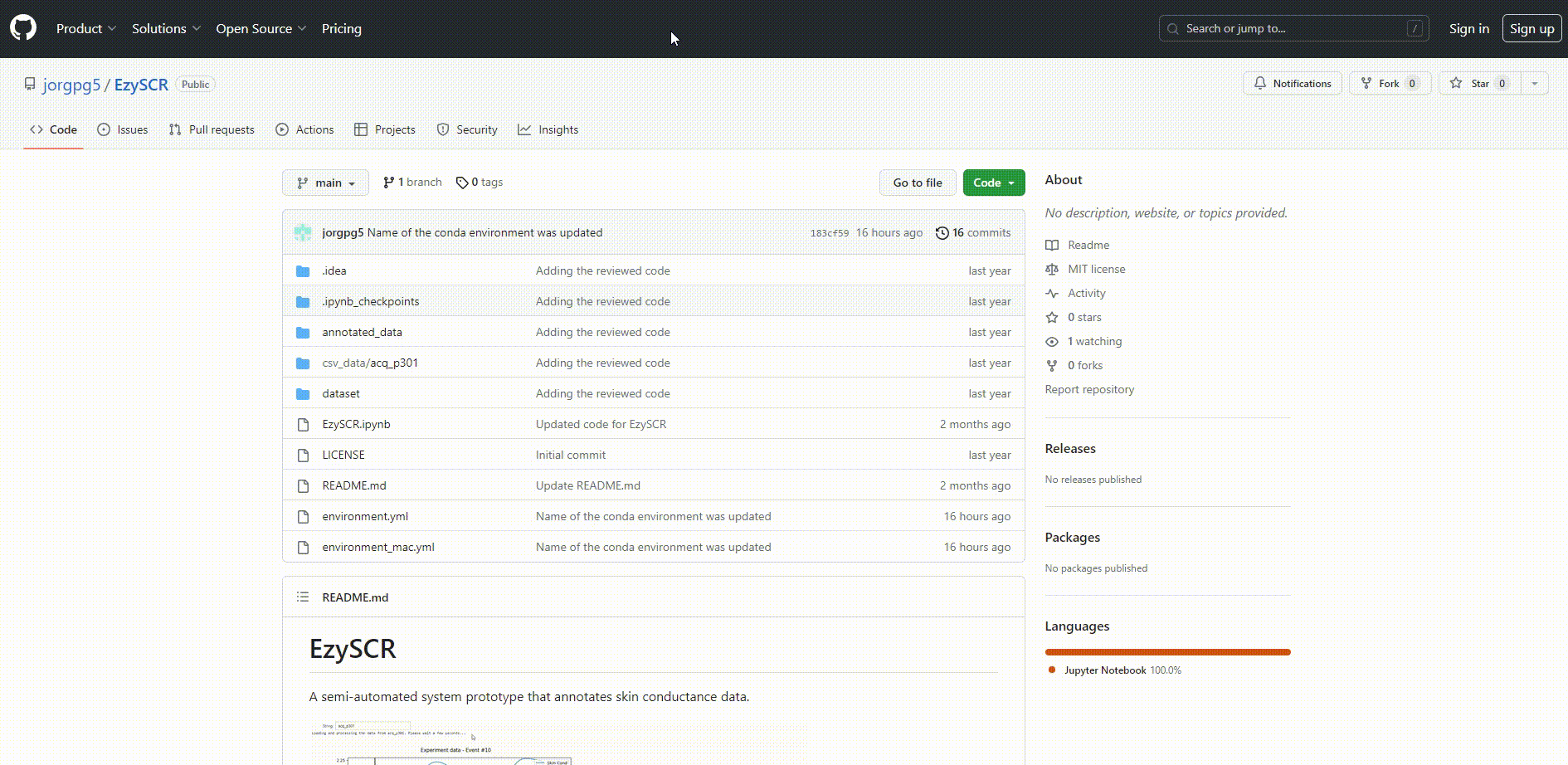


***Figure S5.*** *Copy the Github location*

**5.** In the following step, use the git bash prompt and paste/type this command to clone the repository into the filepath of your choosing (for instance, in the below image the software is saved into the filepath C:/Users/neyl/Documents/semiautomated_annotation, NOTE that EzySCR is already installed in this folder – when you install for the first time it will NOT be visible yet):

git clone https://github.com/jorgpg5/EzySCR.git


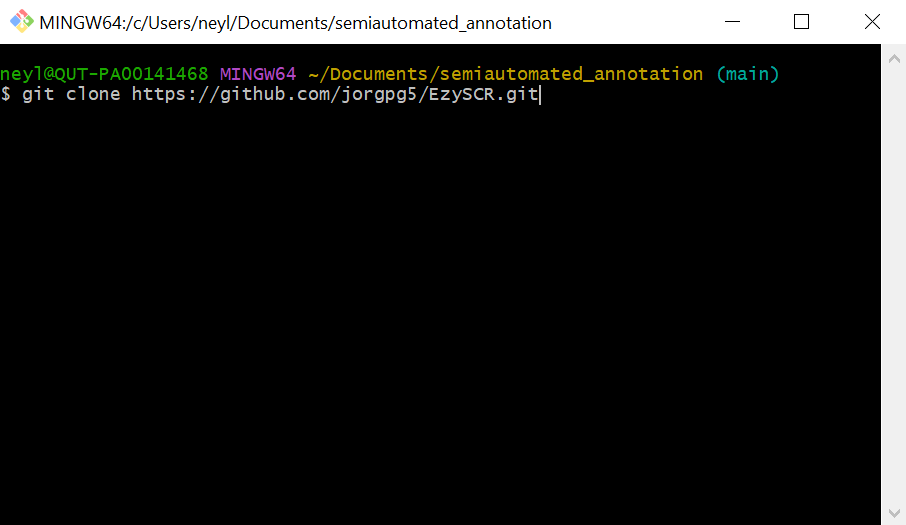


***Figure S6.*** *Clone the git in the folder destination*


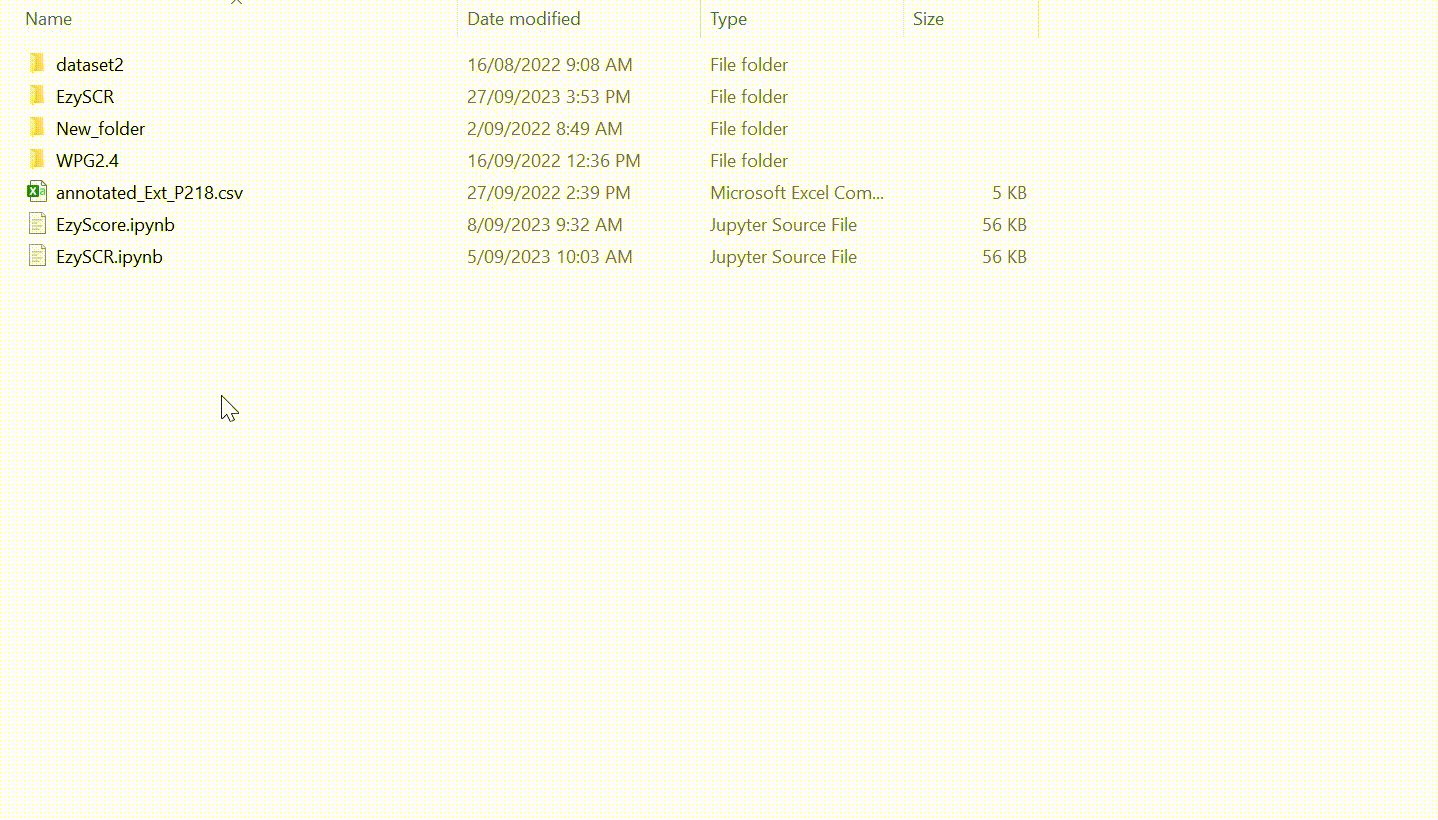


***Figure S7.*** *How to clone the git in the folder destination*

**6.** Then, we will open an anaconda prompt. Type "anaconda prompt" on the windows search bar.

[
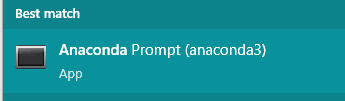
](https://user-images.githubusercontent.com/70129680/182268122-659eb2b1-66e3-4946-919e-689ea0379720.png)

***Figure S8.*** *Open Anaconda Prompt*

We will now install the interface.

**6a.** In the anaconda prompt, go to the folder where you cloned this repository with Git. To set the anaconda prompt to stay at this directory, you can type in the prompt and press enter:

cd filepath

Where filepath is the filepath that directory where you have saved the repository.


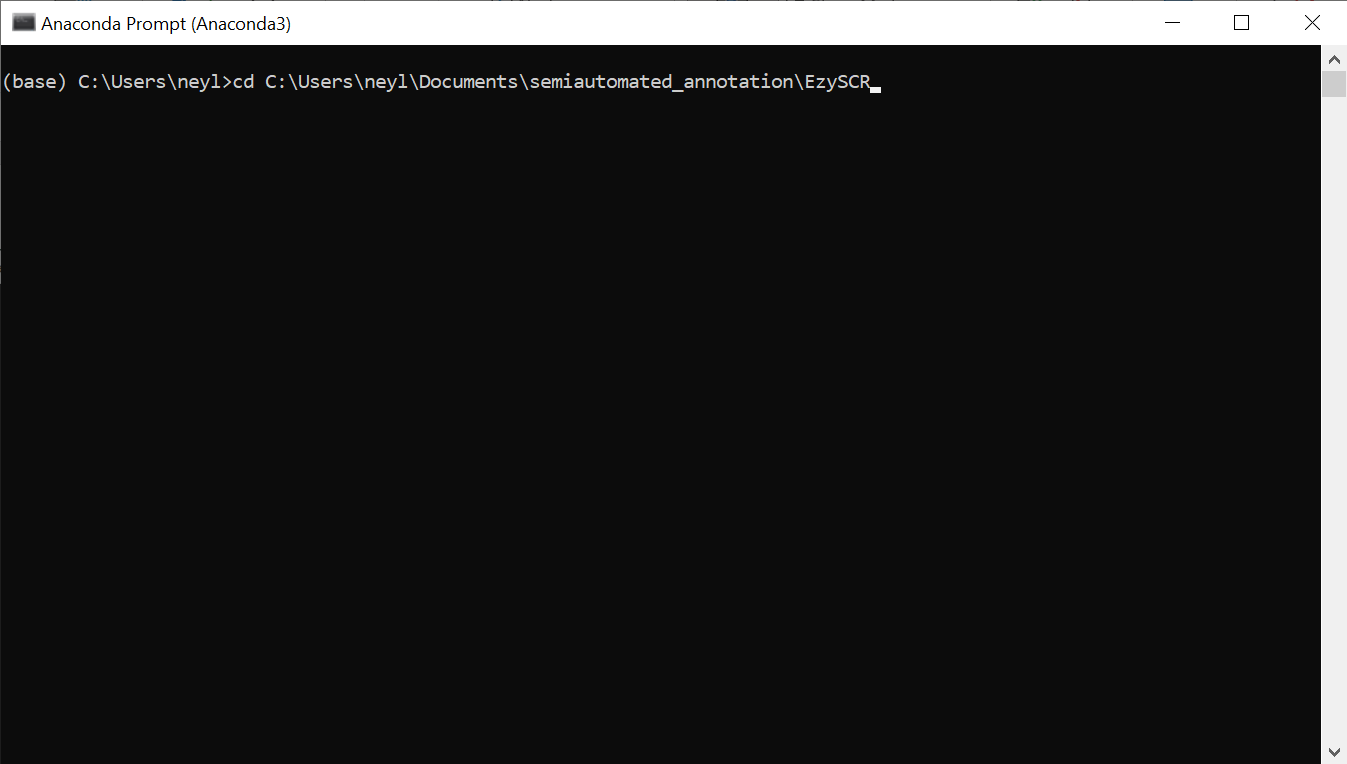


***Figure S9.*** *Change the file path in the prompt window using cd*

### 6b. For Windows:

Type the next command for **windows and press enter**:

**conda env create -f environment.yml**

###
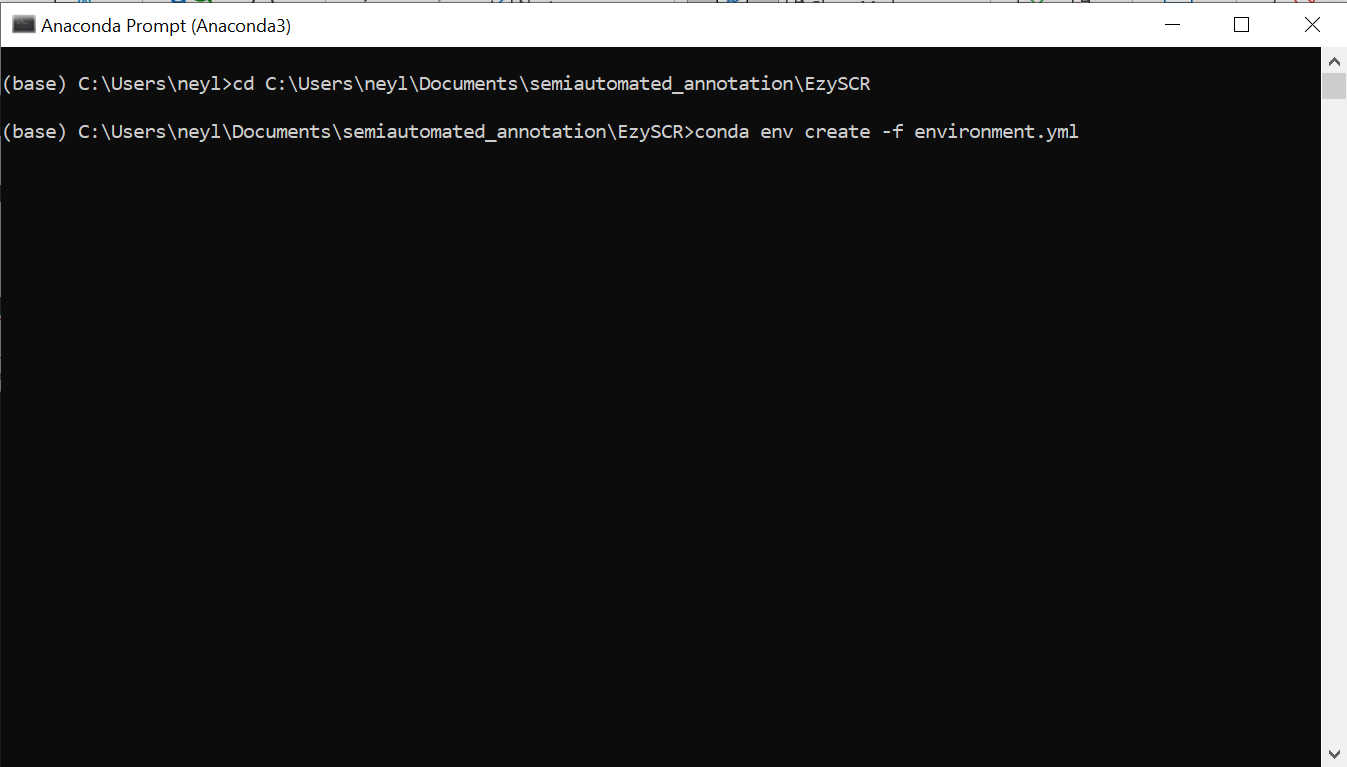


***Figure S10.*** *Type the conda env create command in the prompt window*

### For MacOS

Run the next command for **MacOS**:

**conda env create -f environment_mac.yml**

The virtual environment that hosts the annotation interface should be ready after a few minutes.

Your interface is now ready to use.

**User Guide**

Before you run the interface, you need to double-check a couple of things.

1.- Check that the folder structure is the same as the Git repository.

2.- Check that you have the .mat files in the dataset folder. If you have other types of data files (e.g., Acknowledge .acq or LabChart files), you'll need to use those programs to convert into .mat files by using the Save As or Export functions.

The use of the program is simple, though please refer to the video instructions if you are having trouble: https://youtu.be/YoNKK6LdoH4. To start the program, open the Anaconda prompt and run the command

**conda activate annot_interface**

**
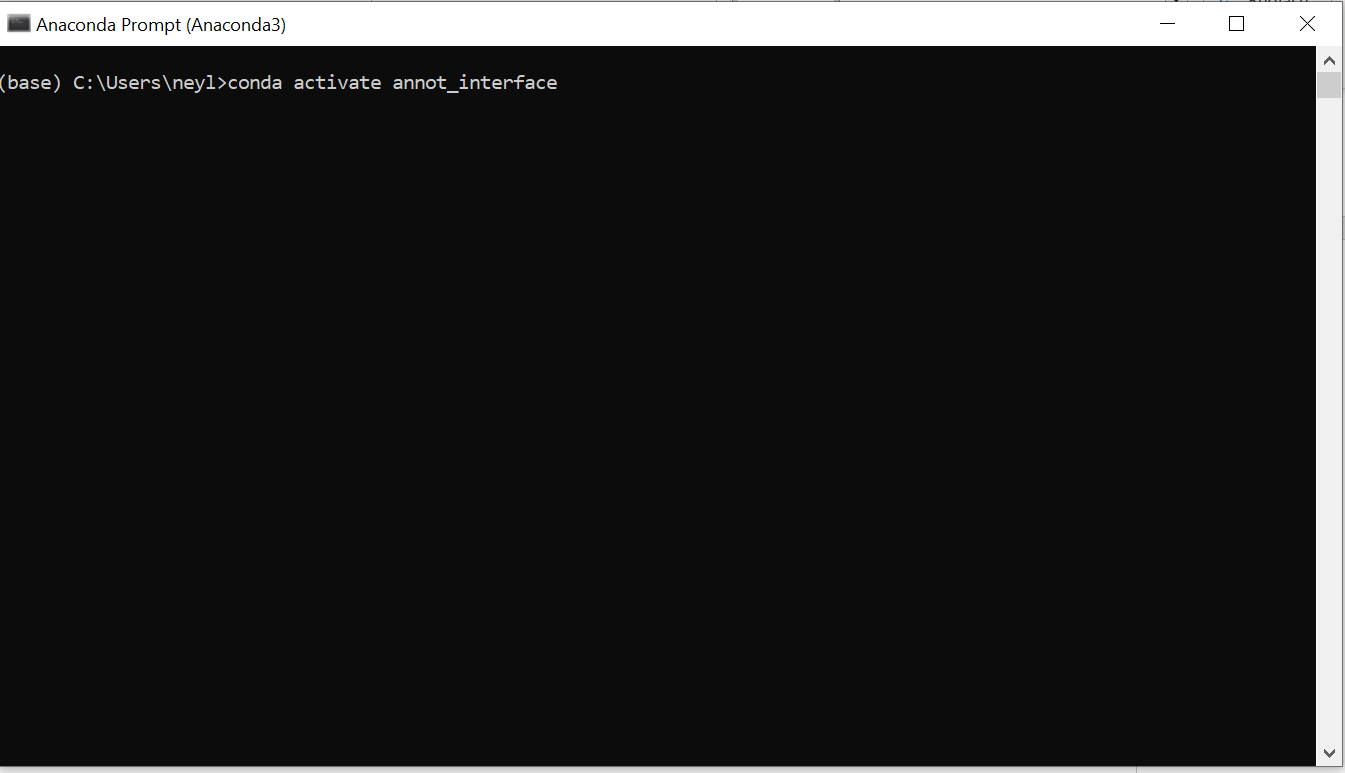
**

***Figure S11.*** *Type the conda activate command in the prompt window*

Next, write the following command

**voila EzySCR.ipynb**

where EzySCR.ipynb is preceded by the file path where it is saved. Again, there must be no spaces in the file path for this step to be successful. If you wish to set your directory to the filepath where the repository is saved beforehand using the cd method described above, then you only need to type **voila EzySCR.ipynb**

**
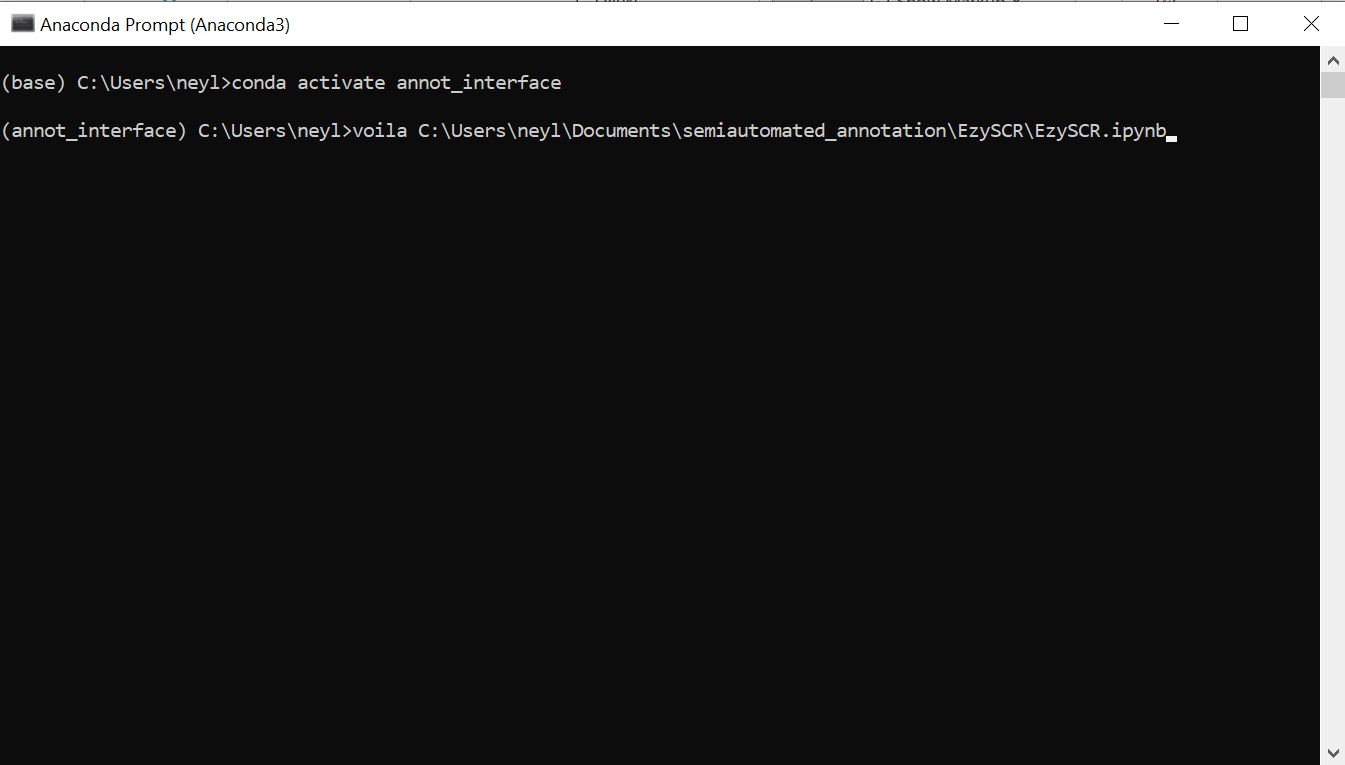
**

***Figure S12.*** *Type the voila command in the prompt window*

After you hit enter, EzySCR will start in one of your browsers. The interface will look like the below image (red numbers inserted after screenshot was captured):


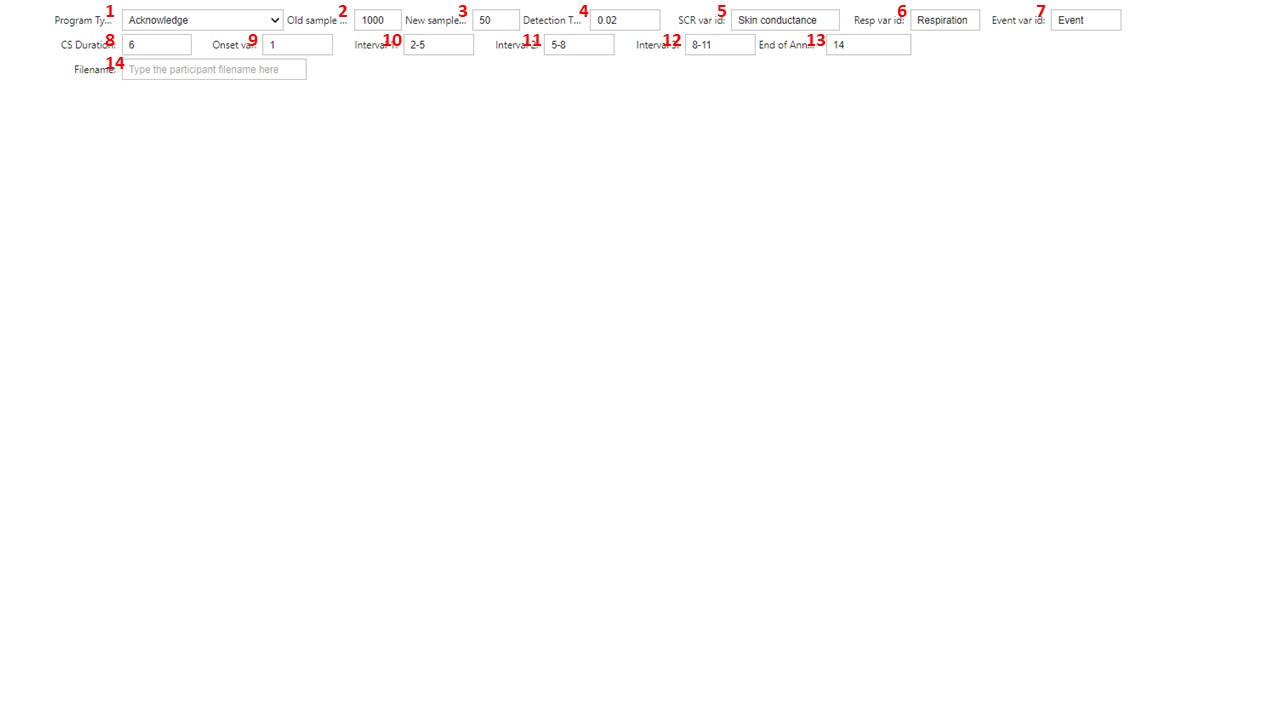


***Figure S13.*** *The current EzySCR options*

Firstly, you need to specify what program your data was recorded in (1), as different programs index their data in different ways. Current Labchart and Acknowledge are supported.

A related general limitation of the software is that it currently only accepts .mat files and it is not possible to immediately import all files from all software programs. Data on SCR software recording programs that we extracted from a recent systematic review (Wang, et al. ^1^, data on software recording programs not published) indicates that over 50% of the fear conditioning field likely uses BIOPAC with Acknowledge software, with many other programs making up smaller shares of the field, including Labchart. In this case, the majority of recorded SCR data will be compatible with the current options available with EzySCR. EzySCR has successfully been tested in two laboratories in North America, one in Europe, and three in Australia.

**If you find that your data is not compatible with the program, please reach out to** [**luke.ney@qut.edu.au**](mailto:luke.ney@qut.edu.au) **and we will find a solution for you immediately. Most issues are due to conflicting formatting of datafiles and are easily fixable by our team.**

You can optionally tell EzySCR what sample rate your data was sampled at (2) and what sample rate you want your data downsampled to (3). The default values are 1000Hz for imported data and 50Hz for downsampling. EzySCR will downsample to 50Hz to reduce preprocessing time, though if you want more (or less) samples in your data then you can change the default setting. *Note:* While increasing the sample rate may potentially improve performance and clarity of results, it can slow the program processing time significantly. If you decrease the sample rate, processing time may decrease but the quality of scores may reduce.

You can also customise the peak detection threshold (4). By default, this is set at 0.02 µS for an inflection in the skin conductance data to qualify as a skin conductance response. You can this to be either larger or smaller.

*Note:* While decreasing the peak detection threshold may increase the number of skin conductance responses that you record during scoring, it may also reduce the efficiency of the scoring algorithm through inclusion of false positive scores.

Each file that you will be processing will include a number of channels where skin conductance data, event (i.e., trial) marker data, and potentially respiration data are recorded. The next step is therefore to tell EzySCR what the Channel Names are. For example, our channel name for skin conductance is Skin conductance. However, this will likely differ for your own laboratory and physiology recordings. Defining the Name of the Skin Conductance (5) and Event Marker channels (7) is compulsory before beginning, and you can optionally register these for a Respiration channel as well (6). If you leave Respiration blank, no respiration data will be included in EzySCR. You can also specify the length of your conditioned stimulus trials in the CS duration box (8). This has a default value of 6 seconds.

EzySCR will score a baseline skin conductance level prior to the onset of the event/trial. This means that, before each event begins, you will get an average skin conductance level. By default, the averaging epoch duration is set to 1 second prior to the event onset (9). However, if you wish to increase or decrease this you can adjust this value. You can also flexibly adjust the duration of the first (10), second (11), and third (12) interval response windows using the available options. The default setting assumes a 6-second event where there is 1 second baseline skin conductance recorded, followed by a 3-second first interval response, a 3-second second interval response, and a 3-second third interval response.

Importantly, note that:

(a) these intervals must be formatted uniformly, i.e., 2-5 or 3-5, etc. (no spaces between, before or after this information).

(b) There must be no gap between the intervals (e.g., 2-5 for first interval and 5-8 for second interval, not 2-5 and 6-8).

(c) Since we specify a baseline skin conductance level interval, whatever you specify here must be added to the first interval latency window. For example, by default Onset_var is 1, which means that the event itself begins at 1 second. In our example, the first interval response will begin 1 second after the onset of the trial and will last for 3 seconds, which is why it is marked as 2-5.

The end of the extracted data for each trial is also specifiable in the End of Ann… parameter (13), which will determine the end of the visualised data that is scorable for each trial. By default, this is set to be 3 seconds after the end of the third interval window.

These defaults are based on a 6-second stimulus event. It is the user’s responsibility to determine which latency window intervals work best for their data. However, typically for stimulus durations longer or shorter stimuli than 6 seconds adjust the second latency window to meet their required length and leave the first and third latency windows at 3 seconds.

Finally, enter the name of the file that you want to analyse (without the extension type, e.g., Acq_P301). If the file is not in the dataset folder, the interface will ask you to verify the filename (10).

Wait a few seconds while the data is preprocessed. Then Click on the "Start Annotation" button that will appear after the file has loaded to EzySCR.


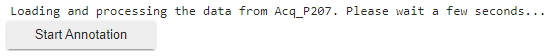


***Figure S14.*** *Press start annotation to begin*

Note that since the program is semi-automated and the peak scores are visualised, no pre-screening of data is necessary (e.g., to filter bad responses). This is done during scoring. Secondly, the interface already has the option to down-sample your data. This means that data does not need to be pre-processed prior to analysis using the program.

The interface will be loaded. The top panels (1) are not interactive and are only a broad illustration of the data across the current event. The bottom panel (2) is an interactive figure that displays the automatically scored responses for the first (3), second (4), and third (5) interval responses. If the automatically scored responses are not accurate, then you can click and drag the red markers on the chart, keeping in mind that skin conductance responses have a trough (6) and a peak (7), and that the peak should always be after the trough. Note that other rules for skin conductance scoring apply, please see Tips and Tricks below. If you're not happy with the annotation, encounter an error, or just want to start over, click on the "Reset" button (8). If you identify an artefact or a non-response that, for whatever reason, cannot be scored, you can select the box (9) below each interval, which will flag the score in a separate column for post-analysis assessment.


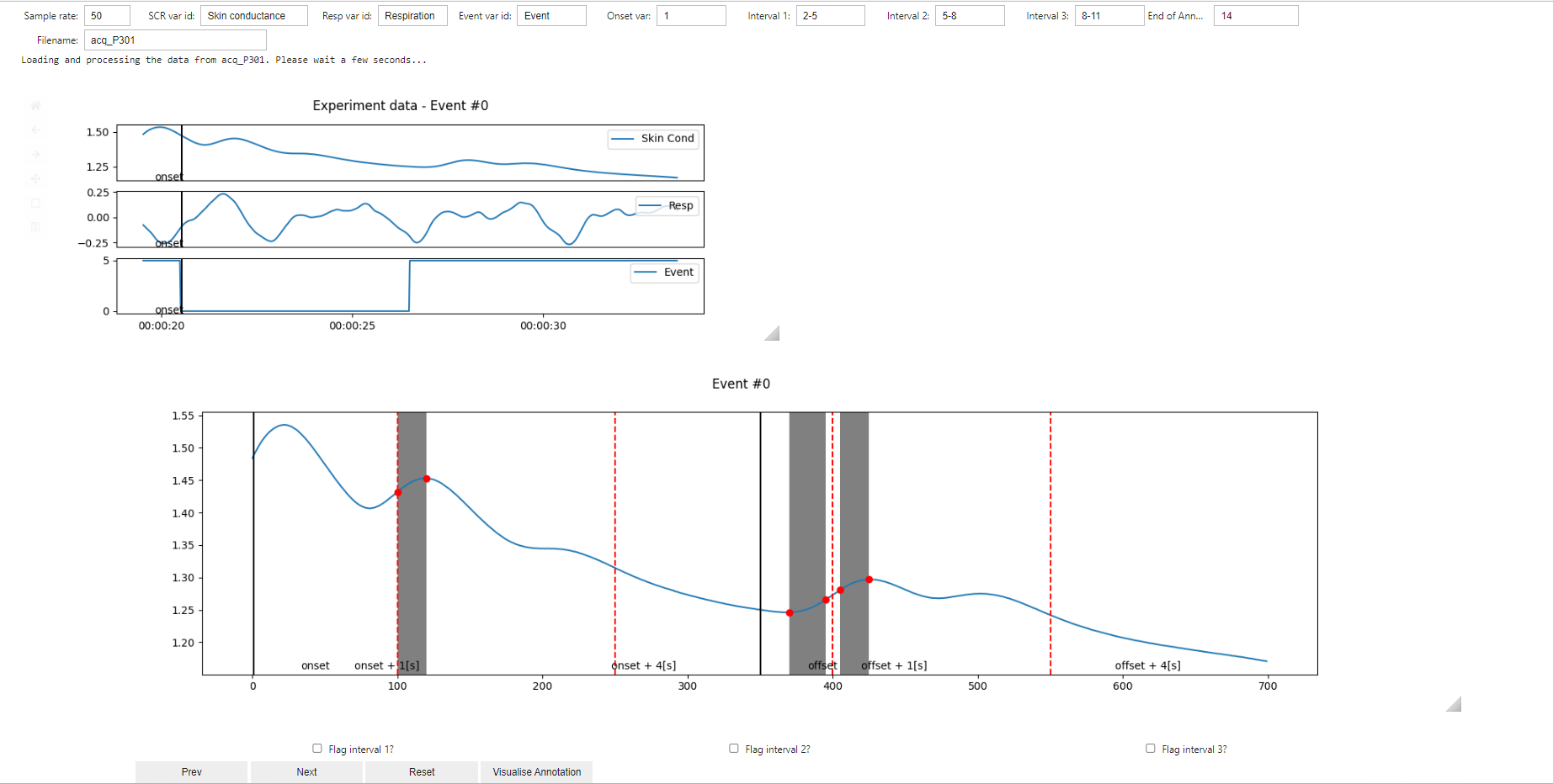


**9.**

**8.**

**7.**

**6.**

**1.**

**3.**

**4.**

**5.**

**2.**

**1.**

***Figure S15.*** *The different features of the EzySCR interface*

To visualise a preview of the data that will be saved, click on "Visualise Annotation". The annotation visualisation reflects exactly what will be recorded in the excel file for that particular trial. This information includes the mean skin conductance level one second before the event onset (1), the peaks and troughs for each of the latency windows (2), and the peak score for each interval, calculated as the difference between the peak and the trough for each interval (3). These latter scores are the ones that will be used for analysis later.


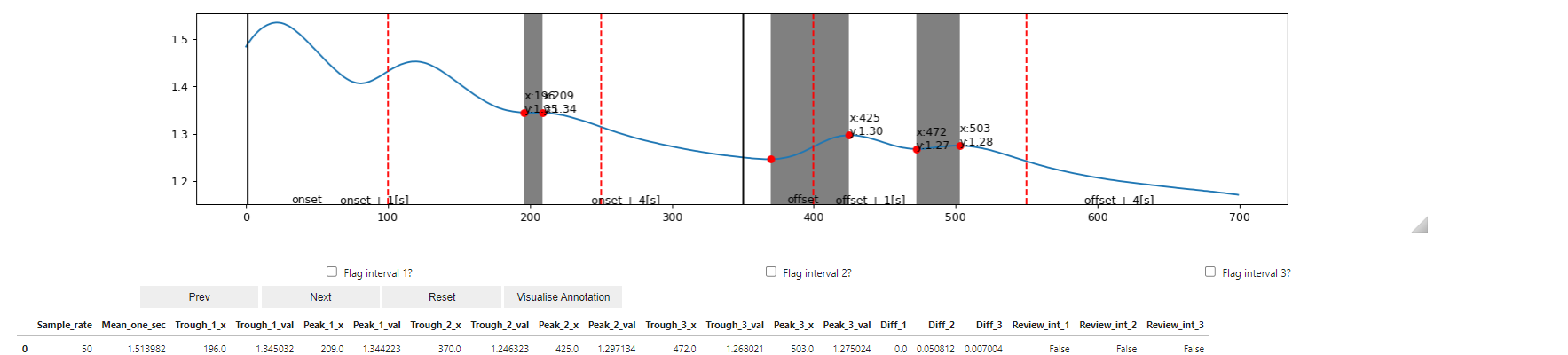


**3.**

**2.**

**1.**

***Figure S16.*** *The EzySCR annotation window*

Click on "Next" or "Prev" to change events. The data will be saved as you progress, such that when these buttons are pressed, the annotations will be saved in the "annotated_data" folder. To make sure that all the data has been annotated, please go through the of all events in that file. Important: If you do not at least look at all of the trials/events in a file, your scored data in the annotated_data folder will not be fully scored.


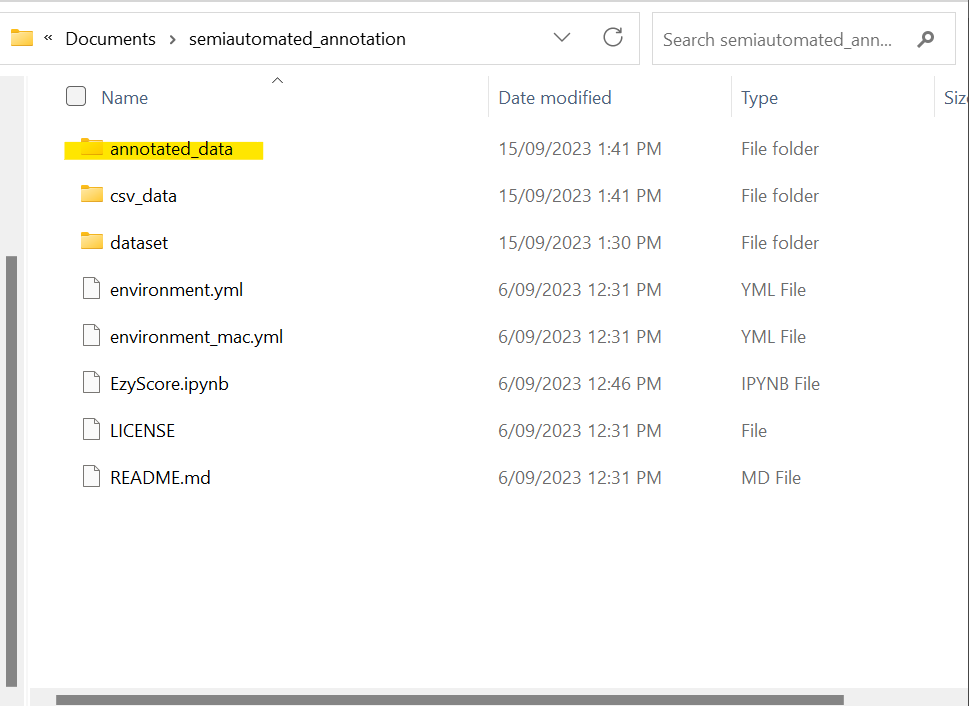


***Figure S17.*** *The file location where your output data is saved*

**After you have finished scoring a file and are ready to load the next file, you should refresh your browser!**

**Tips and Tricks**

The moderate speed of the EzySCR interactive interface is a known issue and is a general limitation of the program. For both Mac and Windows, the best option is to run the program while having your laptop connected to a power source, rather than running on battery power. Running the program while on battery power significantly reduces the processing speed of the program.

Scoring skin conductance responses involve registering trough (i.e., lowest level) and peak (i.e., highest level) scores. This is called Trough-to-Peak (TTP) scoring. In TTP scoring, the trough must precede the peak. For interval-based scoring, the trough must begin within the interval, and the peak can be either within the same interval as the trough, or in a subsequent interval (see examples below).


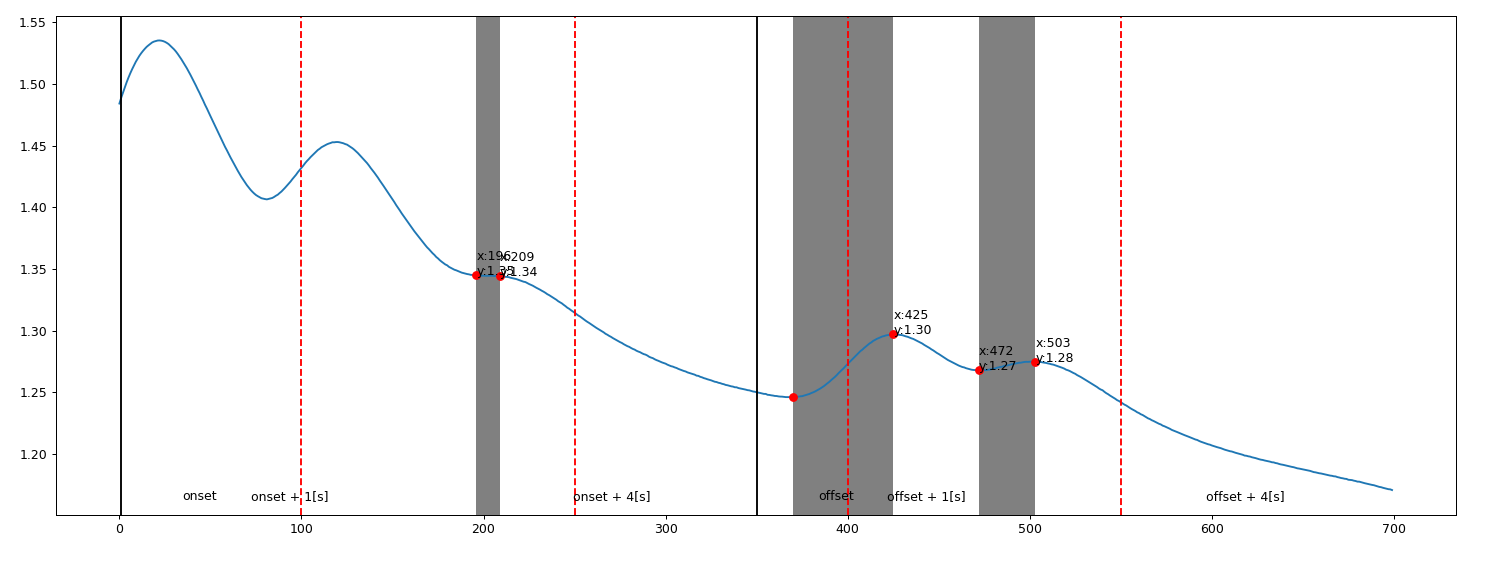


***Figure S18.*** *EzySCR has three scoring windows*

Errors in the EzySCR program can be caused by the user crossing red markers across red markers from other intervals. To fix this error, you will need to reset the event.

The most common source of discrepancy between raters when using EzySCR is disagreement at to in which interval a response begins in. In the first example below, the response begins in the second interval, but in the second example the response begins in the third interval. Ultimately, it is the user’s responsibility to ensure that raters are sufficiently trained to make correct and consistent decisions.


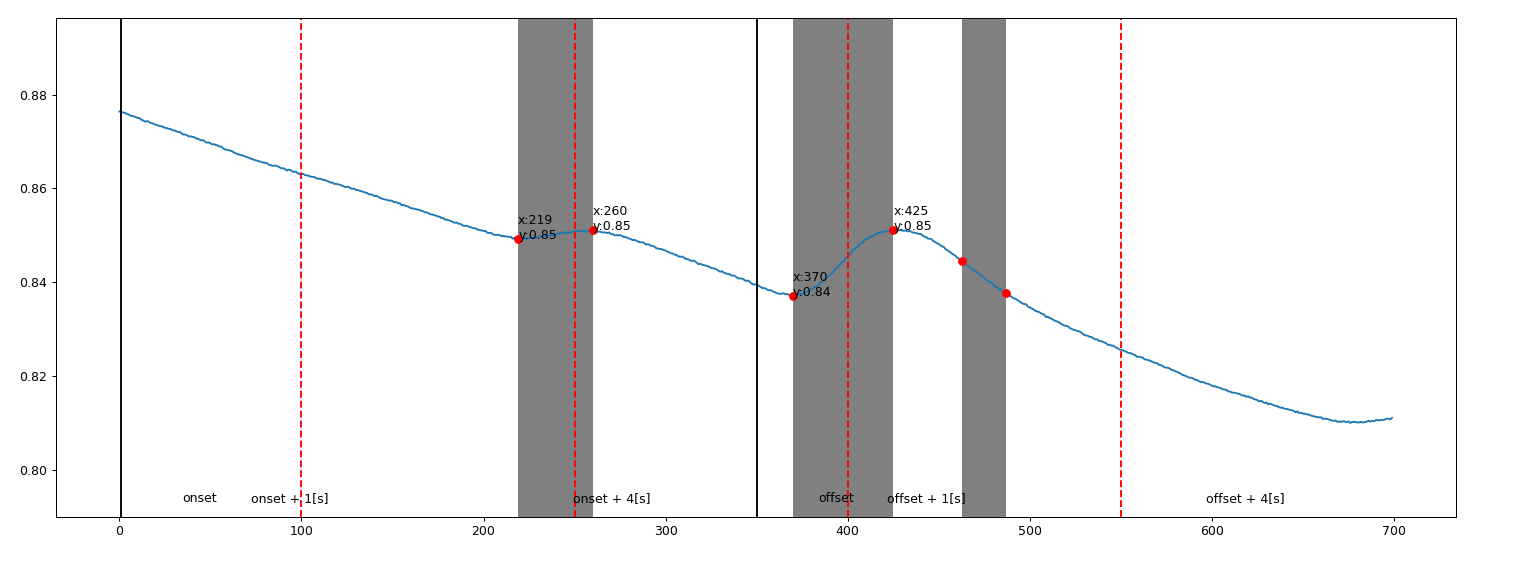


***Figure S19.*** *The second peak here starts in the second interval but peaks in the third interval*


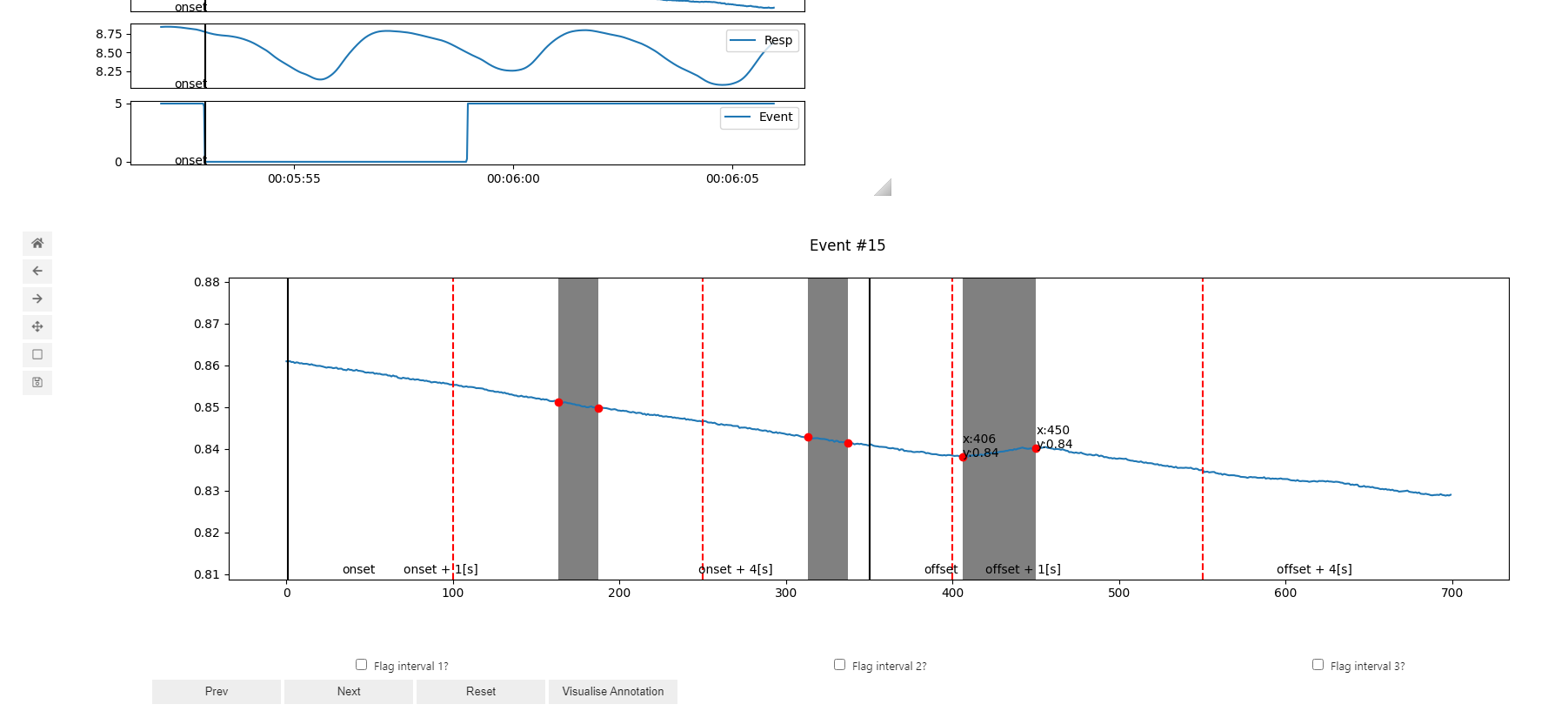


***Figure S20.*** *The third peak here starts in the third interval and peaks in the third interval*

For each interval, the largest response should be scored, if there is more than one response.

One common mistake in scoring skin conductance responses is to add concurrent peaks on top of one another. Studies that use skin conductance usually have long intertrial intervals, because skin conductance takes a relatively long time (e.g., 5 seconds) to return to baseline after a response). This means that, within one interval, you may have two responses that layer on top of each other (see example below), which may look like one response if not considered closely. In these cases, the user should attempt to score only the largest of the two responses. In the example below, the first interval response is incorrectly scored as it should be divided into two peaks rather than one.


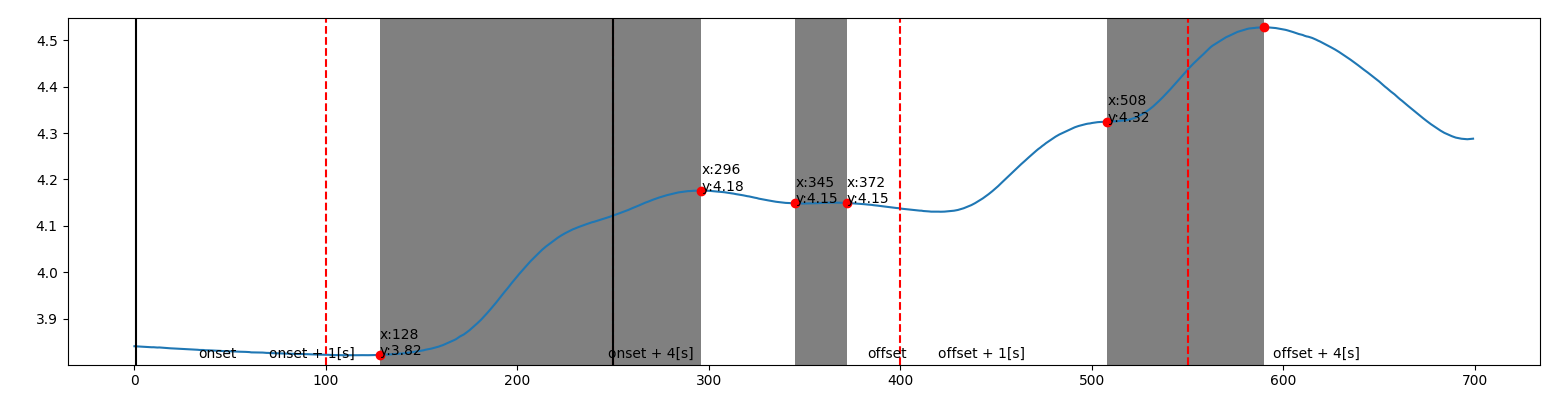


***Figure S21.*** *The first response here is actually two responses*

Often there will be no response in an interval. In EzySCR, this is usually scored accurately by the automatic labelling because skin conductance level tends to decline across time if there are no responses. A correct score for the following case is a trough marker that is higher than the peak marker (see below), as this will result in a score of zero (no negative skin conductance responses are possible). However, in cases where skin conductance level is trending upwards and, in the absence of any peak, a zero score is not possible using this method, the user can select the box below the interactive graph to override the provided score with a zero. This method can also be used if, due to data that is poorly recorded and full of artefacts, a zero response is not possible using the red markers.

**
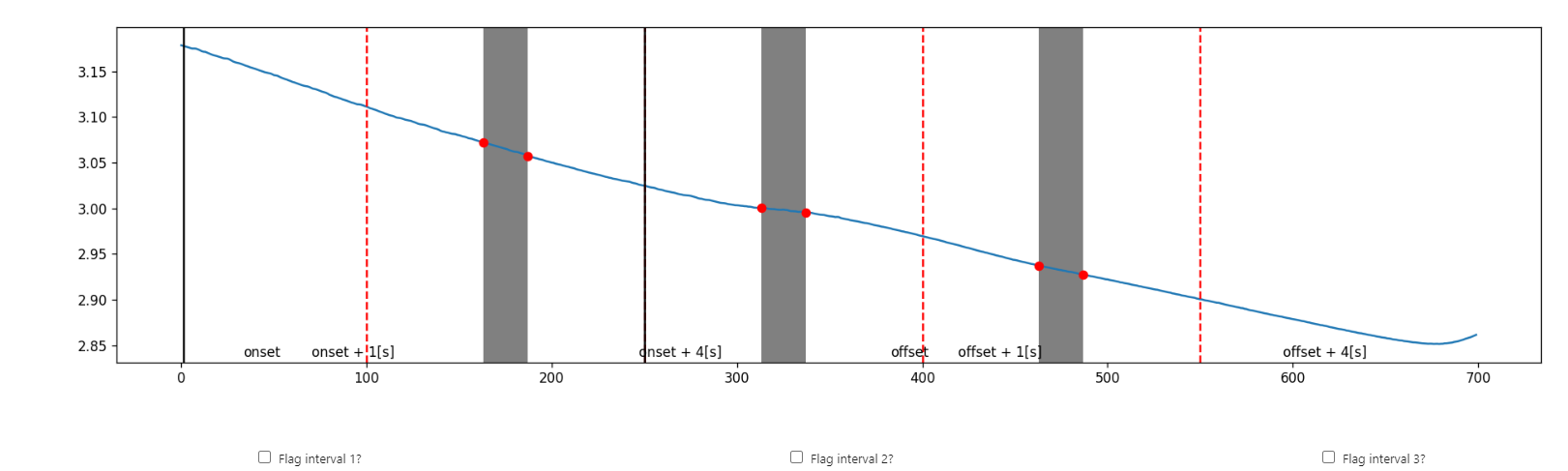
**

***Figure S22.*** *How to score zero responses*

The EzySCR program is very sensitive to the quality of your data. It is the user’s responsibility to ensure that data is properly recorded and that poor quality data is correctly scored. Skin conductance data should always be smooth and the responses Gaussian, with right-leaning skew (i.e., a long response tail).


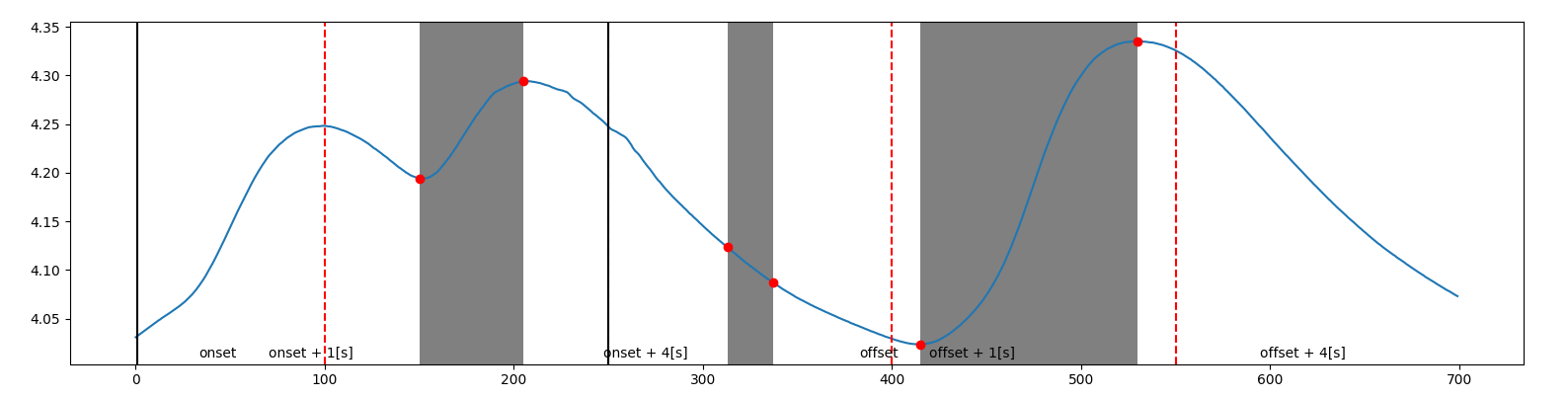
 ***Figure S23.*** *Example of good quality skin conductance responses*

Below are examples of skin conductance fluctuations that should NOT be considered skin conductance responses to the presentation of your stimuli. These examples reflect poor quality data.


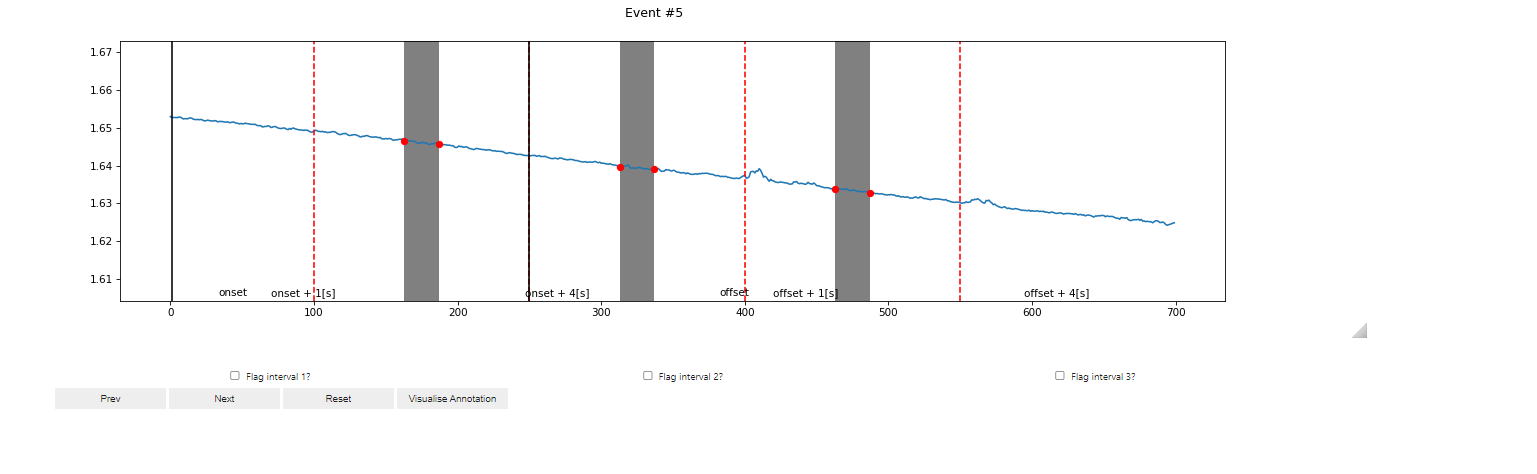


***Figure S24.*** *Poor quality recordings should not be scored as skin conductance responding*

**
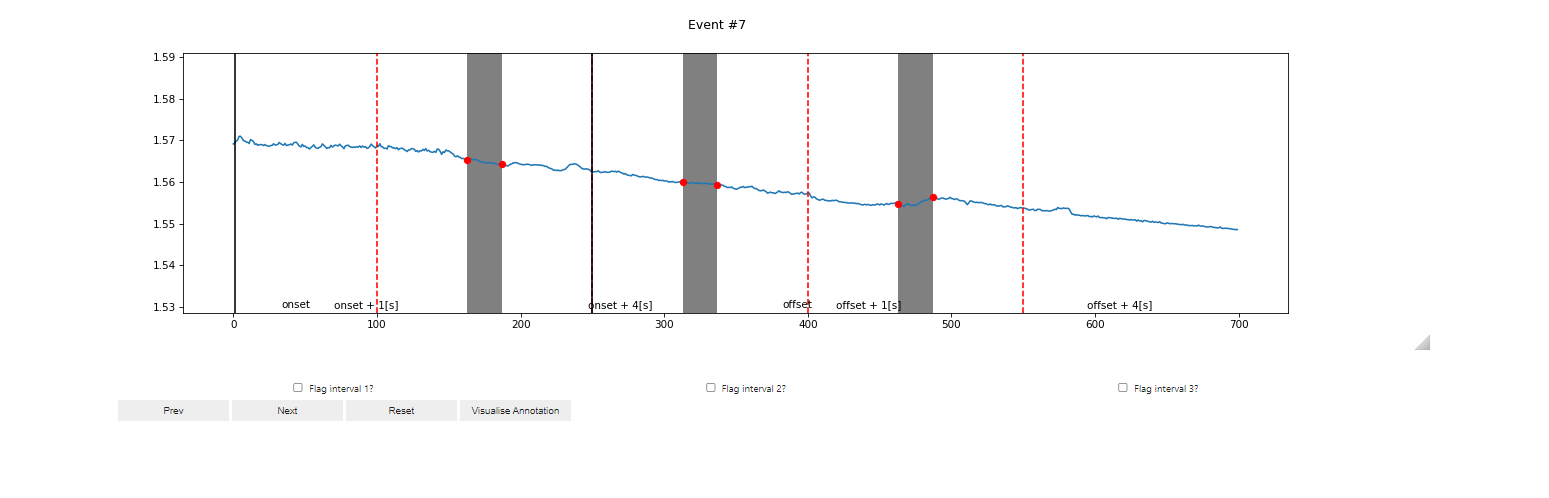
**

***Figure S25.*** *Another example of poor quality skin conductance data*

If you encounter the following error prior to being able to assess your data:


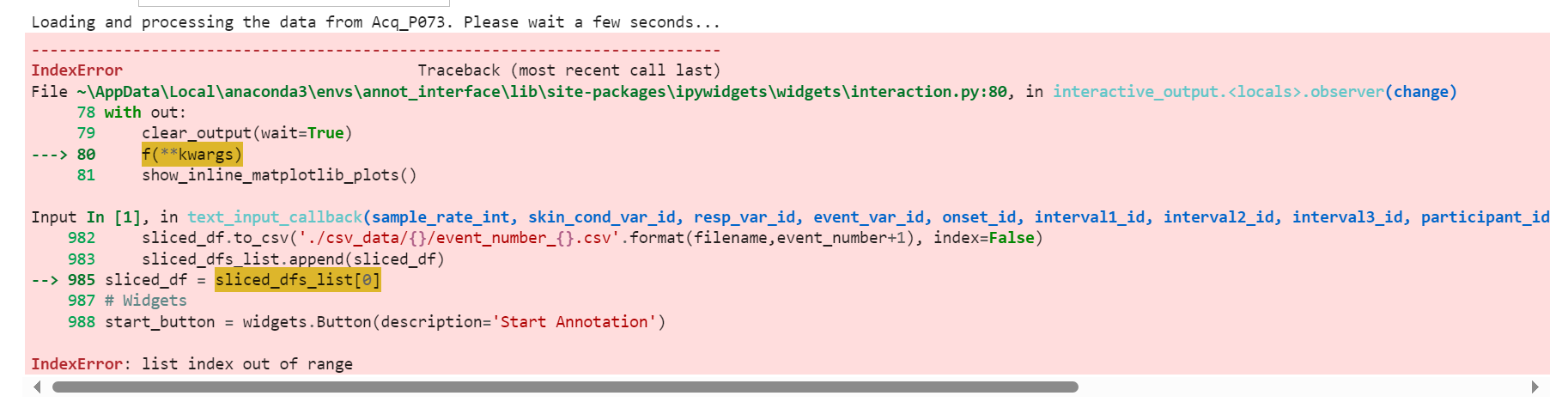


***Figure S26.*** *This error means that your data is not formatted in a way that EzySCR recognises. Contact us for a custom solution.*

This is likely due to your Channel names not being correctly labelled. You will need to ensure that the channel labels (i.e., for skin conductance, event, etc) are labelled in the opening program window exactly as they are labelled in the data file that you are trying to upload.

Alternatively, it could be that your Event channel differs in formatting to what is expected. In this case, please contact us and we can add a specific program type option for your laboratory.

**Contact**

If you encounter issues with the program or have questions, comments or suggestions, please contact Professor Ottmar Lipp ([ottmar.lipp@qut.edu.au](mailto:ottmar.lipp@qut.edu.au)) or Dr Luke Ney ([luke.ney@qut.edu.au](mailto:luke.ney@qut.edu.au)).

The program is regularly updated and we are very responsive to emails!

**Citation**

If you use this software, please include the citation in your manuscript:

Ney, Pardo, & Lipp (2024). EzySCR: A free and easy tool for scoring event-related skin conductance responses in first, second, and third interval latency windows. *Psychophysiology.* Doi: 10.22541/au.171197184.44332055/v1

**References**

Green, S. R., Kragel, P. A., Fecteau, M. E., & LaBar, K. S. (2014). Development and validation of an unsupervised scoring system (Autonomate) for skin conductance response analysis. *Int J Psychophysiol*, *91*(3), 186-193. https://doi.org/10.1016/j.ijpsycho.2013.10.015

IpyWidgets Dev Team. (2015). *ipywidgets, a GitHub repository*. In https://github.com/jupyter-widgets/ipywidgets

Kluyver, T., Ragan-Kelley, B., Perez, F., Granger, B., Bussonnier, M., Frederic, J., Kelley, K., Hamrick, J., Grout, J., Corlay, S., Ivanov, P., Avila, D., Abdalla, S., & Willing, C. (2016). *Jupyter Notebooks – a publishing format for reproducible computational workflows*.

The Pandas Dev Team. (2020). *pandas-dev/pandas: Pandas*. In (Version latest) Zenodo.

QuantStack. (2019). *Voilà*. In <https://blog.jupyter.org/and-voil%C3%A0-f6a2c08a4a93>

Wang Y, Olsson S, Lipp OV, Ney LJ. Renewal in human fear conditioning: A systematic review and meta-analysis. Neuroscience & Biobehavioral Reviews. 2024;159:105606.
